# Modeling of the control logic of a UASS based on coefficient of variation spraying distribution analysis in an indoor flight simulator

**DOI:** 10.3389/fpls.2023.1235548

**Published:** 2023-08-21

**Authors:** Adhitya Saiful Hanif, Xiongzhe Han, Seung-Hwa Yu, Cheolwoo Han, Sun Wook Baek, Chun-Gu Lee, Dae-Hyun Lee, Yeong Ho Kang

**Affiliations:** ^1^College of Agricultural and Life Sciences, Interdisciplinary Program in Smart Agriculture, Kangwon National University, Chuncheon, Republic of Korea; ^2^College of Agricultural and Life Sciences, Department of Biosystem Engineering, Kangwon National University, Chuncheon, Republic of Korea; ^3^Upland Mechanization Team, National Institute of Agricultural Sciences, Department of Agriculture Engineering, Rural Development Administration, Jeonju, Republic of Korea; ^4^Department of Agriculture and Biosystem, Korea Polytechnic, Gimje, Republic of Korea; ^5^Department of Smart Agriculture, Korea Agriculture Technology Promotion Agency, Iksan, Republic of Korea; ^6^Department of Biosystems Machinery Engineering, Chungnam National University, Daejeon, Republic of Korea; ^7^Department of Crops and Food, Jeollabukdo Agricultural Research & Extension Service, Iksan, Republic of Korea

**Keywords:** UASs, indoor spraying simulator, coefficient of variation, nozzle, machine learning, pump opening

## Abstract

**Introduction:**

In the past decade, unmanned aerial spraying systems (UASS) have emerged as an effective crop treatment platform option, competing with other ground vehicle treatments. The development of this platform has provided an effective spraying system that can be used on all crop types and in all weather conditions. However, related research has not been able to develop a UASS that can be operated in windy conditions with a low drift percentage.

**Methods:**

In this research, spraying was simulated in an indoor flight simulator by considering flight speed, altitude, wind speed, wind direction, rotor rotation, interval, spraying pattern, and nozzle type, which were used as the parameters affecting the output value of the coefficient of variation (CV) of spraying. These parameters were referenced as properties that occur in the field, and using machine learning methods, the CV value was used as a dataset to develop a model that can execute pump opening by controlling the flow rate. There are four machine learning methods used, i.e. random forest regression, gradient boosting, ada boost, and automatic relevance determination regression which are compared with simple linear regression and ridge regression as linear regression.

**Results:**

The results revealed that the random forest regression model was the most accurate, with R2 of 0.96 and root mean square error (RMSE) of 0.04%. The developed model was used to simulate spraying with pump opening A, which connects two nozzles in front, and pump opening AB, which connects all four nozzles.

**Discussion:**

Using the logic based on CV value and pesticide quantity, the model can execute the pump opening against the environment and UASS operation.

## 1 Introduction

Plant pests and diseases are the main factors responsible for a significant reduction in crop production, including crop yield and quality (Godfray et al., 2016). Guo et al. reported that pests, weeds, and plant diseases accounted for 30% of global crop losses annually (Guo et al., 2019). Therefore, measures must be taken to reduce the enormous impact of pests and diseases. Currently, the spraying of chemical pesticides on crops is the most widely used method for preventing and controlling diseases and pests (Chen et al., 2021; Sparks and Bryant, 2021; Zhang et al., 2021). Various methods have been developed to improve the spraying efficiency and control the effect of pesticides, such as ground spraying, aerial spraying, air-assisted spraying, and knapsack spraying (Qin et al., 2016; Pan et al., 2017; Wang et al., 2019).

In the last decade, the price of pesticides for plant maintenance has soared (Wilson and Tisdell, 2001). Moreover, the use of pesticides in the past year reached an average of 5–7 liters/ha (Maria Travisi et al., 2006). However, the inappropriate use of pesticides results in a decrease in productivity owing to decreased soil nutrients. In addition, the indiscriminate use of pesticides affects crop yield because pesticides affect soil nutrients, interfering with plant growth and directly affecting crop yield (Tudi et al., 2021). Accordingly, the impact of indiscriminately used pesticides is felt on crops. In addition, the exposure of pests and diseases to large quantities of pesticides results in the development of resistance. In some plant pests, this resistance can be passed on to the 5th–7th generation, increasing difficulties in controlling these pests via chemical control in the next planting periods (Shi et al., 2011). This indicates that the correct application of pesticide-based plant treatments can improve the quality of the plants. In addition, this can affect the productivity and optimal growth of plants on fertile lands (Kalia and Gosal, 2011). This indicates the importance of the appropriate application of pesticides in terms of quantity and accuracy of the needs of plants.

Spraying systems that utilize aerial vehicles have a greater application range and can overcome the negative impacts of pesticide use, as most of these impacts are related to the ground rather than air (Li et al., 2022). Compared to conventional spraying methods, such as knapsack sprayers or ground vehicles, unmanned aerial spraying systems (UASS) exhibit a greater spread distance (Maheswaran et al., 2020). However, as this system operates by flying over the ground, it is susceptible to strong winds, which results in drift. In addition, owing to the maximum light payload and high energy output of the system, the power source, which is a battery, runs out quickly, resulting in a limited operational time. The use of a flexible platform makes it simpler to meet the intended needs. However, this platform encounters problems operating UASS for spraying fields in windy situations. Compared to conventional knapsack spraying techniques and ground vehicle plant protection, UASS exhibits significantly increased operational efficiency with reduced labor costs and pesticide exposure (Zhu et al., 2010). In addition, UASS exhibits numerous advantages, including a higher rate of pesticide penetration into the crop, owing to the ability of the rotors to overturn the leaves (Meng et al., 2019). To date, significant studies have been conducted on the use of plant protection techniques to reduce pests, such as the development of precision UASSs (Zhu et al., 2010; Li et al., 2022), autonomous ground vehicle plant treatment (Maheswaran et al., 2020), and in specific sectors, such as crop protection machinery for vegetables (Wang et al., 2019). Consequently, this has resulted in a significant increase in the use of plant-protection UASS.

The fundamental factor in agrochemical capacity control is the accuracy of the spray target in spray deposition pesticides. The use of a different platform slightly disrupts these fundamental plant protections; thus, it is essential to develop a new spraying system that will comply with applicable regulations. In addition, UASS manufacturers must consider variables that may emerge during operation (Chen et al., 2022; Huang et al., 2023). This is because ignoring these factors may reduce the effectiveness of UASS owing to malfunctions, such as drift (Lan and Chen, 2018; Liao et al., 2019; Hussain et al., 2022). Recently, UASSs have been widely employed in the agricultural sector and combined with cutting-edge technology to meet aerial spraying needs (Chen et al., 2022; Huang et al., 2023). In addition, research findings have been used as feature upgrades to develop new platforms. Typically, UASS developers do not produce upgraded components traded separately from the main platform, and they want to capitalize by increasing the selling value of their products by incorporating research features (Gregorio et al., 2014; Butler Ellis et al., 2017).

Pesticides are typically applied uniformly across the land. Even if not all areas of the agricultural land are infected and require treatment, the treatment requirements of disease-infected plants determine the pesticide dose. As the distribution of pests and diseases determines treatment, the amount of pesticides sprayed is unevenly distributed. In contrast, a plant-targeted protection treatment with the right dose will enable the rapid completion of the process before the disease spreads throughout the land (Bottrell and Schoenly, 2018).

Previous studies have reported several limitations of each UASS category. Operation parameters are the main factors that are still an operational consideration. Rotor rotation is an advantage not exhibited by other sprayer systems (Meng et al., 2020), but should be considered in the operation of the UASS (Lan and Chen, 2018). Some UASS manufacturers advise that they should be operated in a conducive environment. This is because an unstable condition affects the effectiveness of the flexible platform; particularly, the use of the UASS during inclement weather may result in the uncontrollable loss of pesticides. Manufacturers have addressed this weakness by upgrading the system via the development of a new platform rather than the addition of feature-enhancing components, such as new control systems. To overcome the limitations of UASSs, their features must be improved to meet the needs of their users. For optimal performance, not only the quantity of pesticides required but target location requiring a specific treatment should be considered, and these must be fulfilled under any operating conditions or environment, indicating that the system must overcome all negative parameters for the UASS to complete the target as soon as possible before the spread of pests and diseases (Hanif et al., 2022).

The quality of effective spray width and overlap identifies the effectiveness of a UASS spray, as shown in the method in ISO 5682-1 (ISO (International Organization for Standardization), 2017a), and another indication is uniformity. The uniform distribution of pesticides on plants indicates a good spraying distribution and the safety of pesticide penetration on plants (Lv et al., 2019). The spray’ uniformity across the nozzle’s working width demonstrates an even distribution. This context has been demonstrated by the low coefficient of variation (CV), which has been reported that the maximum acceptable level of CV is 30% (Parkin and Wyatt, 1982; Richardson et al., 2004; Richardson et al., 2020). CV value obtained from overlapping between spray lines in the operation of spray distribution (Griesang et al., 2022). Nevertheless, a lower CV indicates a uniform and even distribution in agricultural spraying. The output is expected to meet the goal of spraying as uniformly as possible while using a minimum amount of pesticides.

The most common method for assessing these characteristics is by recording droplet deposition using water-sensitive paper (WSP) and conventional optical techniques to assess the droplet images of the WSP. The WSP can rapidly and easily calculate droplet deposition, coverage, and distribution (Zhu et al., 2010; Cerruto et al., 2019; Brandoli et al., 2021). However, it only qualitatively displays the occurrence of deposition, approximates the droplet size distribution portion, and cannot be used to investigate the dynamic. Cunha et al. evaluated the capability of the imaging systems of WSPs and found that most imaging systems cannot precisely measure the coverage density of droplets when the coverage rate exceeds 17%. Consequently, with the growing concern about the adoption and growth of UASS, it is essential to develop a specific standard method or equipment that can determine the spraying deposition pattern of UASS, either in target or off-target regions (Cunha et al., 2012).

This study conducted UASS tests in an indoor simulator under varied controlled conditions, such as wind effect and UASS operation conditions, focusing on developing a spraying control system for row crops. Data collection as a modeling dataset in the indoor simulator had also been adapted to the layout of row crops in the field, with the same planting distance and planting rows in accordance with the standard planting of row crops. The main objective was to develop a machine learning-based model for spraying distribution and characteristic data using the indoor simulator analysis results. Most studies used indoor simulators to analyze the spray distribution characteristics of the UASS type, nozzle type, or spraying scheme. The specific objectives of this study were: 1) analyzing UASS operational conditions, such as flight speed, flight height, rotor rotation, and even spraying pattern, and environmental factors, such as wind condition, as the parameters that need to be considered during UASS operation and assign their values as condition variations, 2) conducting simulations using indoor flight simulators under various conditions to analyze the spray distribution characteristics, and 3) modeling the control system logic using the dataset generated by the simulation in the mission to obtain a uniform spraying distribution.

## 2 Materials and methods

### 2.1 Prototype instrumentation

#### 2.1.1 Main platform (UASS)

**Figure 1:**
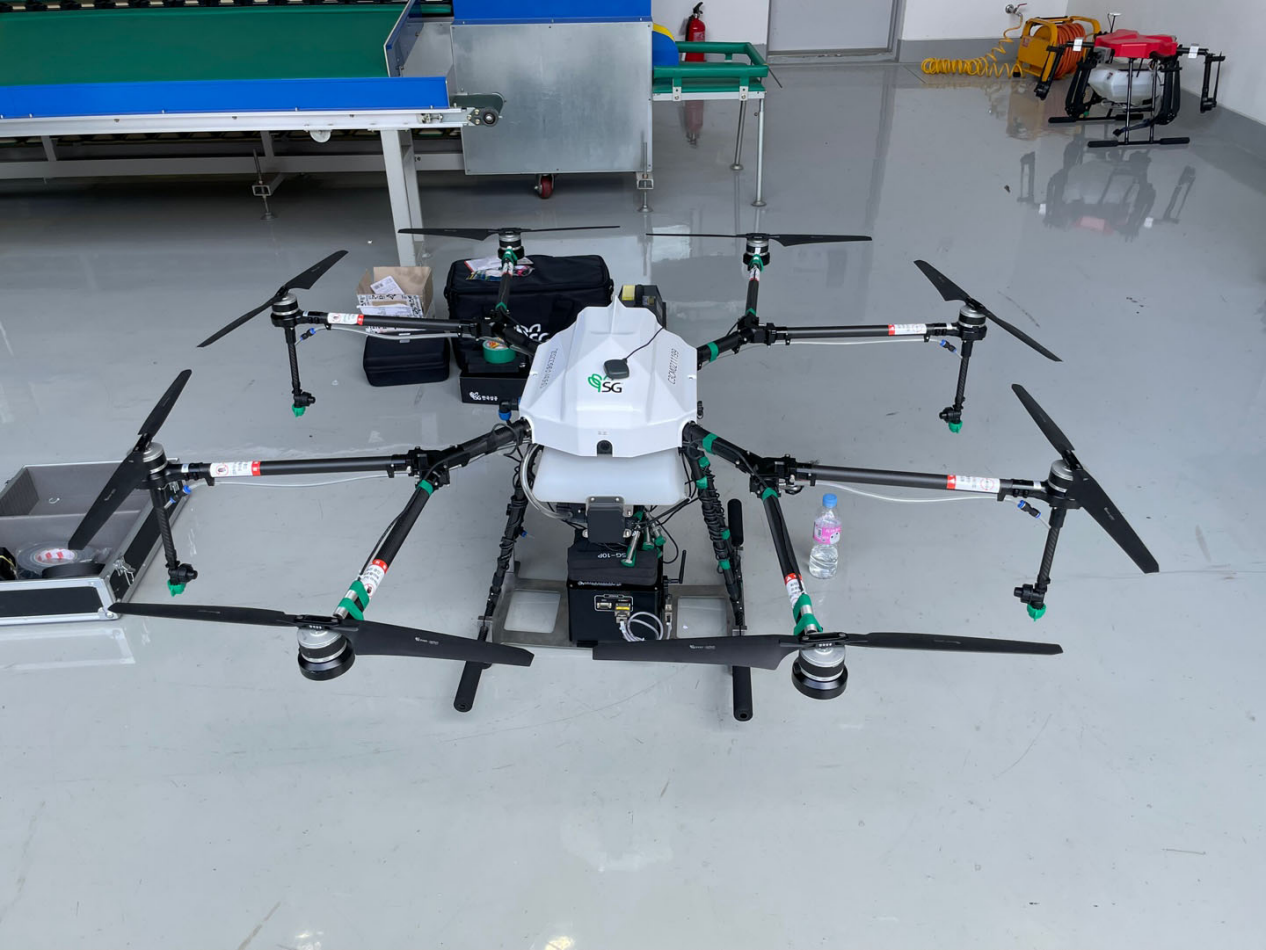
SG-10P UASS used as a prototype.

The control system model in this study was developed using a Korean octocopter UASS platform. The SG-10P (Hankook Samgong Co. Ltd., Seoul, South Korea) UASS was used, as shown in **Figure 1**. This UASS employs four nozzles with an under-main rod rotor system rather than a series configuration like a boom sprayer. The unfolded dimension of the 8-rotor UASS is 1500 mm (length) × 2075 mm (width) × 700 mm (height) with a propeller diameter of 57.5 cm and a spray tank volume of 10.3 L. The system’s net weight (excluding the battery) is 10 kg, and the maximum take-off weight is 24 kg. Four nozzles are mounted below the four lateral rotators of the UASS with a horizontal spacing of 150 cm.

This nozzle placement enables spraying by collectively operating the front and rear nozzles separately. The tank capacity (10.3 liters) will affect the rotor rotation speed in spraying applications. This is because the volume of pesticides in the tank, which decreases with the operation, causes the adjustment of the rotor rotation to the same flight altitude. Increasing rotor rotational speed is the same as operating the UASS at a specific flight speed. The rotor rotation will increase with an increase in the platform’s flying speed setting. In addition, the pitch angle of the platform on the SG-10P increases with an increase in the flying speed.

#### 2.1.2 Type of nozzles used in UASS

Although the main body of the platform does not affect the spraying application, the nozzle type contributes to a high spray quality. Alternatively, this study selected AI series nozzles that have been modified and developed (Rural Development Administration, Jeonju, South Korea) and XR series nozzles 110015 type (TeeJet Technologies, Glendale Heights, USA) were selected. **Table 1** shows the specifications of the two types of nozzles used.

**Table 1:** Specifications of the AI and XR series nozzles used in developing the control system.

Nozzle type	Specifications	Figure
AI series by RDA	• The optimal spraying pressure is between 4 and 8 bar. • The AI nozzle has a spraying angle of 80° which is good at overcoming drift. • It has a finer droplet size at high pressure • More capable of overcoming drift with larger droplet sizes.	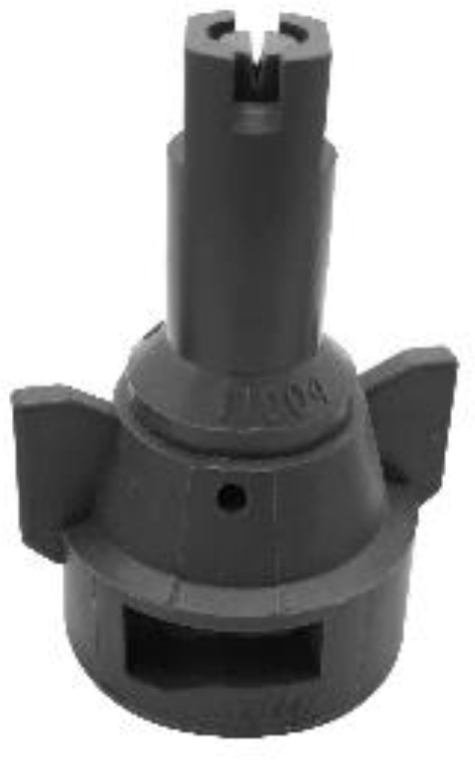
XR series by TeeJet	• It has excellent spray distribution over a wide range of pressures 15-60 PSI (1-4 bar). • The XR nozzle has 110° spray angles • Reduces drift at lower pressures and better coverage at higher pressures. • An excellent nozzle for Pulse Width Modulation Nozzle Control	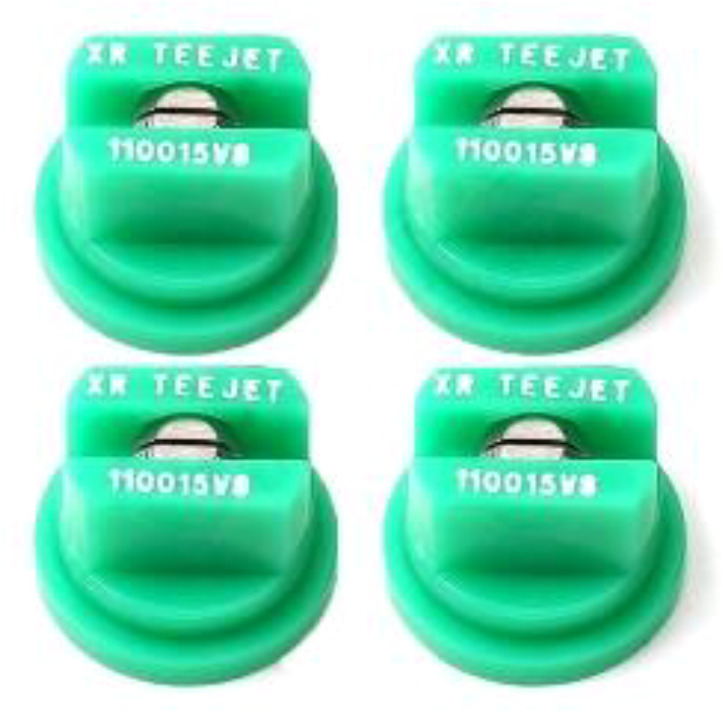

The AI series nozzle can overcome drift but sprays larger-sized droplets, making crop penetration difficult. The XR series nozzle exhibits flexible specifications in terms of spray pressure. This nozzle type can better cope with drift when used in UASS for low-altitude spraying. Using the XR series nozzle at a high pump pressure enables smoother droplets and improved coverage. These two types of nozzles were used to investigate the possibility of using different types of nozzles in UASSs. These two nozzles are an option if the user uses a nozzle type with similar specifications.

### 2.2 Indoor simulator

The UASS performance data can be retrieved in two ways: 1. The direct application of the system in the field or land, but this method has the disadvantage of random and uncontrollable parameter values. 2. An indoor flight simulator was used in this study (Korean Agriculture Technology Promotion Agency, Iksan, South Korea; **Supplementary figure 1**). The indoor flight simulator enables the adjustment of the parameter values according to the needs of various conditions. The total operating conditions used in this study combined all operational and environmental parameters. The combined variations also included two types of nozzles and two types of pump openings, where pump opening A opens two nozzles connected at the front, and opening AB opens all four nozzles at the front and rear.

#### 2.2.1 Spray distribution recording device

**Figure 2:**
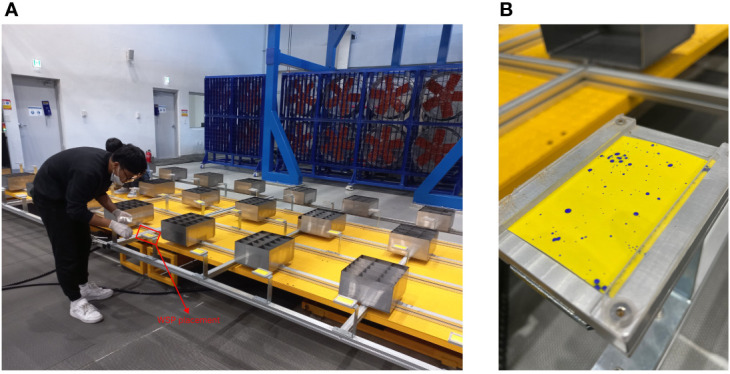
The use of WSP in recording spray distribution, **(A)** by installing WSP on the WSP placement layout, and **(B)** is one of the spray results from the AI series nozzle.

The most standard device for measuring the spread of plant treatment sprays was TeeJet water-sensitive paper (WSP) (TeeJet Technologies, Glendale Heights, USA). The WSP was layered with a yellow film, which changes to a dark blue color upon contact with droplets owing to the interaction of the bromophenol blue indicator on the surface of the WSP with water (Fox et al., 2003; Zhu et al., 2011). The area of the WSP that changes color indicates the deposition quantity (Semião et al., 1996; Sies et al., 2017). The primary constraint of the WSP is the inability of droplets of diameter below 50 mm to create a measurable stain (Semião et al., 1996; Hoffmann and Hewitt, 2005; Mahmud et al., 2016). The size of the WSP used was 50.26 mm × 76 mm, which can resolve droplet diameters of approximately ~30 µm. According to ISO 5682-1 (ISO (International Organization for Standardization), 2017a), measurements can be performed with devices that have an equal surface area, and in this study, WSP was used as a measuring device instead of petri dishes. In **Figure 2**, the use of WSP as a spraying distribution recorder was utilized in the indoor simulator by placing it in WSP placement as shown in **Figure 2A** and **Figure 2B** shows the spray result recorded on WSP from one of the simulations of AI series nozzle.

#### 2.2.2 UASS attachment devices

The indoor simulators used in this study are suitable for obtaining controlled datasets required for model development with machine learning methods. Although the UASS platform can be used directly in the field, the parameter values will vary according to the weather conditions during the tests. Therefore, the prototype platform was installed in an indoor simulator to get controlled parameter values. The main component where the UASS was placed in the simulator is the UASS attachment. The UASS attachment in the indoor simulator can fit on all types of UASS because it uses four combination rods that can rotate 360°, making it universal for various kinds of UASS. The four rods connected the main rod of the UASS to the simulator using a different-sized manufacture jig for each UASS.

The main jig functions to install and connect the UASS with the simulator. The diameter of the jigs matched that of the SG-10P main rods; four jigs were installed on each connecting rod simulator, which then adjusted the degree of rotation of the connecting rods. Owing to the nature of the universal simulator, which can be used on all types of UASS, this simulator also adjusts the system type of each UASS. The simulator can move forward and backward using a rail track whose speed can be adjusted through command control, and according to ISO 5682-1 (ISO (International Organization for Standardization), 2017a), the test shall be carried out with the spraying system moving towards the horizontal spray area, which is in accordance with the test method. In addition, considering that this type of UASS exhibits a different kind of flight system, with some of them adding speed by tilting the pitch angle, as well as increasing rotational speed, this indoor simulator can adjust the degree of roll, pitch and yaw tilt, each of which can be adjusted with paired UASS features. The UASS was installed in this indoor simulator by considering the operation and making the working direction perpendicular to the WSP, which was placed above the ground adjuster.

#### 2.2.3 Ground height and fan generator adjustment

The height of the UASS spraying was not adjusted directly on the UASS attacher, but through the ground adjuster. This component enables the moving up and down of the distribution value recording device (i.e., WSP) and the adjustment of its height according to the operating altitude of the UASS. To consider wind in the simulation, the simulator was equipped with a wind generator with three fans of two different sizes. In **Supplementary figure 2**, the fan in the center position has six blades, and the two fans on the sides have four blades, and the blowing direction of all fans can be adjusted by sliding with the equipped rolling wheels. First, the device must be calibrated to determine the maximum speed at 100% performance to adjust the wind speed. The calibration process was performed using a wind effect measurement system, which also records the value of the direction of the gusts. After calibration, the calibration scale was used to set the desired wind speed.

#### 2.2.4 RPM recorder

The rotation of the rotor or propeller needs to be considered in the operation of the UASS because the resulting fluid dynamics are also one of the factors that affect the quality of the spraying distribution (Chang et al., 2023). Rotor rotation data was retrieved in two stages. The first stage was the estimation of the number of rotors rotations in rotations per minute (RPM) as a function of the flight speed, and the second stage was the rotor rotation value, corresponding to the reduction of tank capacity. Later the two will be correlated with each other because if the user uses a certain flight speed, the rotational speed when spraying will decrease as the UASS operates; for example, if the maximum rotational speed obtained when the UASS flies at 3 m/s is 3000 RPM, the speed reduces with a decrease in the tank capacity. The primary function of testing the flight speed against the rotational speed of the rotor is as a reference for the initial value for the linearity equation obtained from the second stage of the test.

### 2.3 Simulation process

#### 2.3.1 Determination of the variable values

The properties of each parameter should be determined before the simulation is conducted in the indoor simulator so that the settings can be adapted to the parameter platforms in the simulator. However, some parameters, such as flight speed and altitude, can be set directly in the simulator through the command control. The wind speed parameter must be set through the command control, as shown in **Supplementary figure 3**, with the calibration scale that has been performed. In addition, this command control also functions in signaling the simulator to start the simulation, stop the simulation, and emergency stop if an incident occurs in the simulation process.

**Figure 3:**
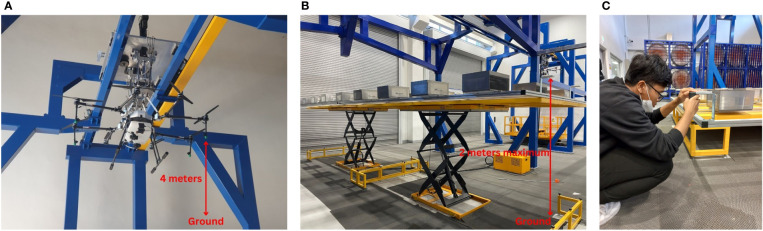
Ground measurement to adjust the spraying altitude; **(A)** maximum distance of nozzle tip to the ground, **(B)** distance of WSP placement to the ground, **(C)** distance measurement process using laser distance stabilizer device.

The flight altitude in the simulator was not achieved by adjusting the UASS’ height but by adjusting the height of the ground where the WSP was placed. The adjustment of the ground height was enabled by the hydraulic rod supports and the regulation of the height of the WSP placement. No precision measurement was provided on this hydraulic rod, so manual measurement using a laser rangefinder was performed. **Figure 3** describes setting the operating spray height by measuring the distance between the WSP placement and the nozzle tip on the UASS. The flying height measurement was obtained from the total distance of the nozzle tip to the simulator floor minus the distance from the floor to the WSP placement. The distance between the nozzle tip and the simulation floor was 4 m, and the maximum distance from the WSP placement to the floor was 2 m, so the minimum flight height that could be achieved was 2 m.

Furthermore, to measure the parameters of the wind effect, the determination and setting were performed in the simulator, but first, the performance calibration of the wind generator was performed. The fan operating performance settings were performed through command control by specifying the percentage value of fan performance. **Supplementary figure 4A** shows a mini weather station used to calibrate the wind speed value generated by the fan. The primary function of this device was to capture wind properties, such as wind speed and direction, and this tool must be set to face the fan perpendicularly. Thereafter, the wind speed and direction values will appear and be recorded, as shown in **Supplementary figure 4B**. The values were recorded for ten variations to get a calibration value where the results obtained are 30% generator performance producing a wind speed of 2 m/s and 60% performance producing a speed of 4 m/s.

Another factor of the platform condition that affects the quality of spraying distribution is the rotor rotation condition that directly produces fluid dynamics at the bottom (Qin et al., 2016). This area is where the pesticide comes out of the nozzle towards the target crop. Thus, in addition to the wind blowing in the environment, the downwind produced by the UASS itself is also influential and must be addressed. Therefore, in this study, the rotor rotation factor was considered as one of the influential parameters.

Each UASS exhibits a different rotor rotation mechanism. Still, most UASS products use a higher rotor rotation mechanism combined with an increased pitch angle, directly proportional to the increased flight speed. In addition, during the spraying operation, the weight of the UASS will decrease as the pesticide is sprayed. This kind of UASS mechanism is similar to the theory outlined by González and Garanger, where the number of UASS rotor rotations will be directly proportional to the payload of the platform, and according to ISO 5682-2 (ISO (International Organization for Standardization), 2017b), the liquid should be measured by the volumetric degradation during the operation of horizontal surface spraying (González et al., 2011; Garanger et al., 2020). These two factors need to be processed to be used as one of the parameters, namely by determining the linearity between rotor rotation with flight speed and tank capacity. Thereafter, the value inputted into the system depends on the flight speed used; then, the rotor rotation parameter can be used with the theory of linearity against tank capacity.

#### 2.3.2 Prototype installation in the indoor simulator

**Figure 4:**
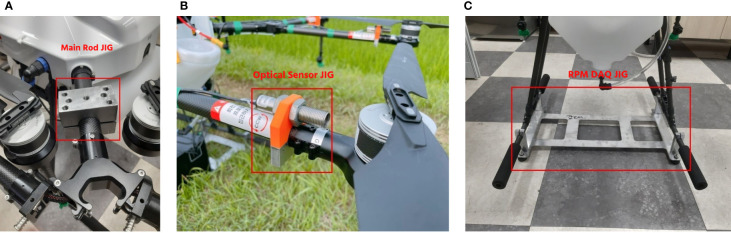
Various jigs used to modify the SG-10P: **(A)** main jig, **(B)** optical sensor jig, and **(C)** RPM DAQ system jig.

There are supporting components used to install the platform in the simulator. As shown in **Figure 4**, the SG-10P was modified as a UASS simulator using three types of jigs. **Figure 4A** shows the jig type that unites the UASS with the simulator with four jigs attached to each main rod of the SG-10P UASS. **Figure 4B** shows the jig supporting the optical sensor ROS-HT-W-25 (Monarch Instrument, Amherst, USA) that sends rotor rotation data to the data acquisition (DAQ) system, and **Figure 4C** shows the jig used to mount the RPM DAQ system (Rural Development Administration, Jeonju, South Korea).

**Figure 5:**
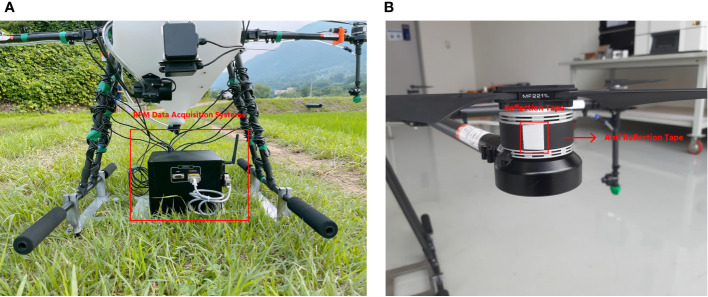
Set of devices used in obtaining RPM data from SG-10P UASS: **(A)** DAQ system and **(B)** placement of reflectance on the rotor for optical sensors readability.

The rotor rotation parameter was determined separately regarding the tank capacity and flight speed. A rotor rotation value was determined using a device that can record rotor rotation. Four optical sensors were placed on two rotors on the front side and two rotors on the rear side to calculate the number of rotations per minute. **Figure 5** shows the RPM recorder device used in the preliminary test with reflection tape attached to the rotor to reflect the light that will be captured by the optical sensors. The RPM recorder was installed inside the UASS during data collection, so the tank capacity data collection accumulated 2 kg. To collect the rotor rotation data as a function of the tank capacity, the number of RPM was calculated at different tank capacities from empty to filled (maximum of 10 liters). Additional data were collected on the rotor speed as a function of the flight speed. Flying speeds of 2, 3, and 4 m/s were used, and this data was taken when the UASS tank was empty. The relationship between the rotor rotational speed against the tank capacity and flight speed was derived to obtain the equation used in the system in real time, and the tank capacity and flight speed were used to determine the RPM parameters.

### 2.4 Parameter analysis and simulation output

#### 2.4.1 Schematic combination of parameter variations

Several factors need to be considered by farmers before operating the UASS in terms of weather and platform conditions. The selection of the performance of the UASS features was also prepared before spraying. A flight speed value of 3 m/s is sometimes used by farmers, so this value was used as the middle value in this study, so that the variation values were 2, 3, and 4 m/s, and the altitude value was set at 2, 2.75, and 3.5 m (Zhang et al., 2021). Thereafter, the wind speed value was set at 0, 2, and 4 m/s with a change in the angle of incidence of the wind to 0, 22.5, and 45°. The rotor rotation used in this study followed the previous data collection and determined the variation value of 2900, 3100, and 3300 RPM. The predetermined parameters were combined scientifically using the orthogonal matrix method. Using five parameters and three levels in this study enabled the extraction of the most effective combination data by the orthogonal matrix so that the simulations performed do not overlap each other and with the maximum amount. The combination of parameters can be seen in **Table 2**.

**Table 2:** Combination of parameters with three levels randomized by orthogonal matrix.

Flight Speed (m/s)	Altitude (m)	Wind Speed (m/s)	Wind direction (˚)	Rotor rotation (RPM)	Result code
2	2	0	22,5	2900	11111
2	2	2	0	3300	11223
2	2.75	0	45	3300	12133
2	2,75	4	22,5	3100	12312
2	3,5	2	45	3100	13232
2	3,5	4	0	2900	13321
3	2	0	45	3100	21132
3	2	4	22,5	3300	21313
3	2,75	2	0	3100	22222
3	2,75	4	45	2900	22331
3	3,5	0	0	3300	23123
3	3,5	2	22,5	2900	23211
4	2	2	45	2900	31231
4	2	4	0	3100	31322
4	2,75	0	0	2900	32121
4	2,75	2	22,5	3300	32213
4	3,5	0	22,5	3100	33112
4	3,5	4	45	3300	33333

#### 2.4.2 Image processing, coverage, and coefficient of variation analysis

The combinations used in the setup of each device produce parameter values in a controlled environment. Using these conditions, the simulation was first performed using one of the nozzle types. One simulation run was performed for each combination of conditions, and the coverage output, which the WSP recorded, was simultaneously generated. To process the simulated WSP to obtain the coverage data, an image processing device was used to read the spray distribution on each WSP sheet. **Supplementary figure 5** visualizes a set of tools that process WSP data into coverage values on each sheet. The attached camera processes the WSP spectrum indicated with water to calculate the number of droplets. The coverage value can be obtained by comparing the sprayed area on the WSP.

**Figure 6:**
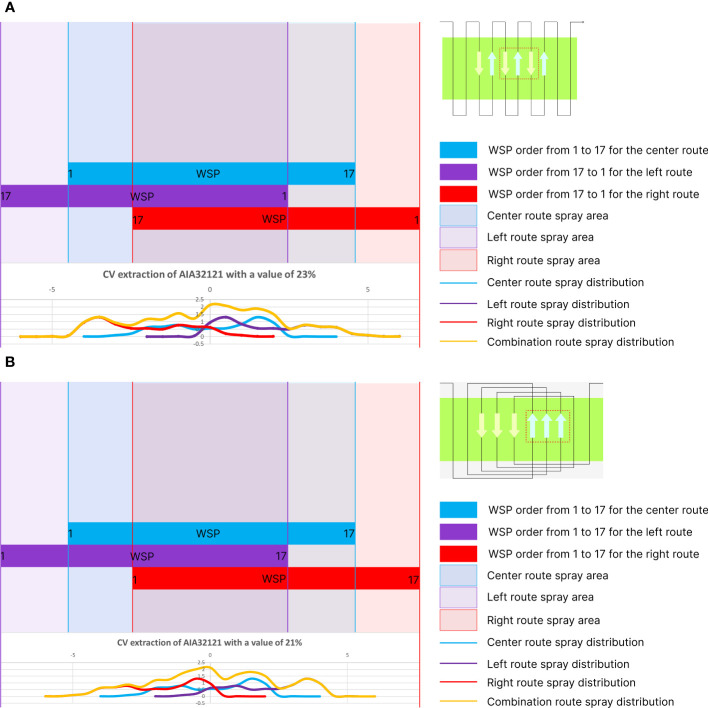
Processing coverage values into CV using: **(A)** back-and-forth and **(B)** race track spraying patterns.

The coverage value data analyzed was extracted into CV values using two types of spraying patterns. The average result of the three rows of WSPs had output coverage values at the effective swath width. In **Figure 6**, seventeen WSP sheets were used to record the coverage value of one of the simulations, and the order of the WSPs represents the spraying route from the bottom to the top. The CV value calculation was done with three lines of spraying routes in one interval distance; thus, in **Figure 6A**, the CV value was calculated using the same WSP arrangement with a race track spraying pattern with the same spray route direction. However, in **Figure 6B**, back-and-forth has the opposite direction on both sides. Based on the back-and-forth spraying rule (Carvalho et al., 2020), the WSP arrangement order can be rotated vertically to indicate the spray direction from top to bottom. Thus, CV values were calculated using coverage values with the order of values reversed on both sides.

The uniformity of pesticide spray distribution is one of the essential factors of pesticide application quality, which is indicated by the CV. The smaller the CV value, the more uniform the droplet distribution and the better the spray quality. The calculation formula is given in Equations (1) to (3) according to ISO 5682-3 (ISO (International Organization for Standardization), 2017c) as follows:


(1) $$\bar{\text{a}}=\frac{1}{\text{n}}\sum_{\text{i}=1}^{\text{n}}a_{i}$$

(2) $$\text{s}=\left[\frac{1}{\text{n}-1}\sum_{\text{i}=1}^{\text{n}}{\left(a_{i}-\bar{\text{a}}\right)}^{2}\right]$$

(3) $$\text{CV}=\;\left|\frac{\text{s}}{\bar{\text{a}}}\right|\times 10$$

Where $\bar{a}$ is the average coverage value at the effective swath width (in %), *n* is the number of effective swath widths, and *a_i_* is the coverage value at the working width range (in %). Thereafter, *s* is the standard deviation of each coverage mean, which is then divided by $\bar{a}$ to get the *CV* value (in %).

The influence of parameters used on the CV obtained through the indoor simulator should be validated, and the validation was performed using analysis of variance (ANOVA), which aims to determine the validity of each parameter used in obtaining the CV value.

### 2.5 Development of spray control system model

The main objective of the simulation was to obtain the CV under various spraying system operating conditions. The variation of conditions determined was in line with the simulation rules, namely using an orthogonal matrix design that enables these combinations to cover all conditions of the various levels specified. Thereafter, this output data was used as the dataset to determine what treatment should be executed to provide a uniform spraying distribution if values are outside the variation of conditions.

In this study, two methods were used to develop the prediction model for the operating state of the UASS: the first method uses linear regression, and the second method is machine learning. Two types of linear regression methods were selected, namely simple linear regression and ridge regression, and four types of machine learning (random forest regression, ada boost, gradient boosting, and automatic relevance determination regression (ARDR)). The R^2^ and root mean square error (RMSE) values of these two methods were compared. During the development of the model, 70% of the dataset was used for calibration and 30% for validation.

The number of datasets used in calibration significantly affects the accuracy of the resulting model. The ability of the model to find a regression function to predict the situation improves as the dataset of variation results increases. Therefore, the datasets obtained during the simulation were combined by giving the identity of each output so that they were not mixed up, considering that the same conditions were used in two types of nozzles. The datasets of the AI series nozzle and XR series nozzle were merged by representing the AI nozzle with number 1 and XR with number 2. In addition, the datasets were merged on the spraying pattern used in processing the coverage value into CV, namely back-and-forth represented with number 1 and race track with number 2. After combining the nozzle types and spraying patterns, the total dataset for pump opening A was 648, and 648 for pump opening AB. The pump opening here is an option that will be the actuator in the control system, where pump A operates the front nozzle and pump B operates both rear nozzles. The control system has actuators with openings A and AB; therefore, the two datasets obtained from the simulation of different openings cannot be merged for modeling. These two pump openings will have different equations and be used as a logic model in determining which pump opening (A or AB) is better used in determining the real-time operating state of the UASS.

## 3 Result

### 3.1 Equation of RPM parameter

The combination of the flight speed, altitude, wind speed, wind direction, and RPM parameters are shown in the orthogonal matrix (**Table 2**). The three levels of each parameter were determined by taking the value often used by farmers in operating the UASS as the center value, followed by the two upper and lower border values. The rotor rotation value was measured to calculate the RPM of each rotor at different flying speeds and tank capacities.

**Figure 7:**
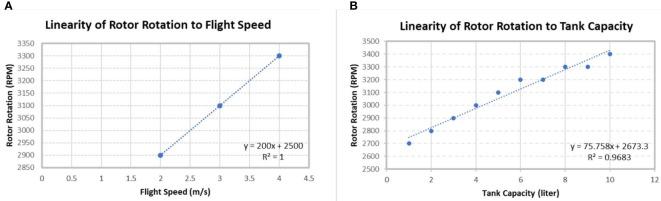
Linear graph of the relationship between **(A)** flight speed and rotor rotation and **(B)** tank capacity and rotor rotation.

Two types of RPM data collection methods were conducted in the preliminary test to determine the parameter values. The first test involved calculating the rotational speed with a change in the flight speed, both of which exhibited a linear performance (**Figure 7A**): the rotor rotation value increases with an increase in the flight speed. The rotor rotation values were calculated using the resulting linearity formula value at speeds of 1 to 5 m/s at an increase of 1 m/s for data processing purposes.

In addition, it is essential to understand the relationship between the rotational speed and tank capacity, as the number of rotor rotations is directly proportional to the weight of the UASS. The results revealed that the rotor revolutions decreased with a decrease in the tank capacity to adjust the flight altitude. Under full tank conditions where the UASS is in maximum payload, the resulting rotational speed was 3400 RPM, which exceeds the rotational speed of the specified maximum flight speed parameter of 3 m/s (**Figure 7B**). Therefore, the RPM parameter was derived using the tank capacity and flight speed.

The data for the two variables (flight speed and tank capacity) were processed using the statistical analysis software Minitab (ver. 20.3, Minitab, LLC., State College, USA) application to obtain equation linearity, as expressed in Equation (4) as follows:


(4) RPM=2574.3+122.2v+88.36m 

Where RPM value is determined by *v* as flight speed (*m/s*) and *m* as tank capacity (*liter*), which is multiplied by a constant value, and an interception value is given, which was derived using the regression method, the R-squared value was 87.77%, indicating that the model was quite valid and can be used.

Thereafter, this equation was used as one of the parameters in the system control, where the flight speed and tank capacity can be input in real time so that the calculation can be performed as the UASS operates.

### 3.2 Comparison of the spraying distribution under different nozzles and spraying patterns

**Figure 8:**
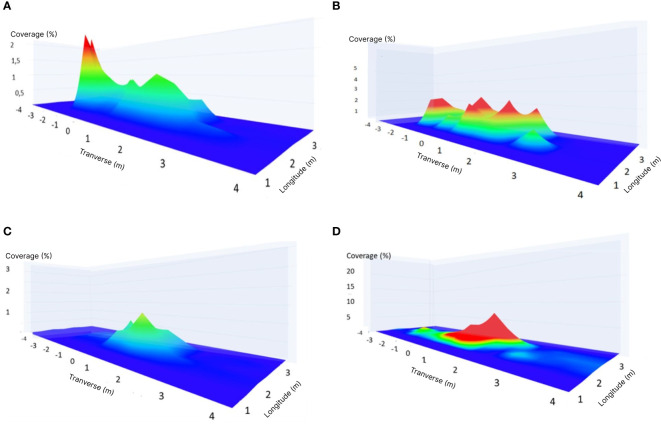
Mapping of the spray distribution under general conditions with **(A)** AI series pump opening A, B. AI series pump opening AB, **(C)** TeeJet XR series pump opening A and **(D)** TeeJet XR pump opening AB.

Simulations were performed using the indoor flight simulator based on all the predetermined parameters. The combination with code 32121, primarily used by farmers, was analyzed at a flight speed of 4 m/s, an average height of 2.75 m, no wind effect, and an average rotor rotation of 2900 RPM. In **Figure 8**, the coverage value was obtained from a simulation where the UASS moved and operated the spray over the three rows of WSPs arranged on the layout.

The characteristics of the AI nozzle, which can spread further and spray large-diameter droplets, are shown in **Figures 8A, B**, where both A and AB openings exhibit a wide spray spread of up to 6 m. At pump opening A, the spread on the left side indicates a coverage value of above 2%, which can be attributed to the anomalies of nozzles. The spray distribution results of the TeeJet XR series nozzle in openings A (**Figure 8C**) and AB (**Figure 8D**) revealed that the uniformity of the distribution was high at a distance of 1–1.5 m from the center point. The shape of the distribution was consistent with the spray mechanism: there was a more significant amount at the center owing to overlapping from both sides of the nozzle. The coverage of pump opening A at the center point was approximately 1%, and that of opening AB was 10%. There was an anomaly with pump opening AB, in which water that should be sprayed actually drips by the time the UASS reaches the spray point, resulting in a significant increase in the diameter of the droplets that fell on the WSP and an increase in the coverage to 10%.

#### 3.2.1 CV value of race track spraying pattern at 1–9 m intervals

**Figure 9:**
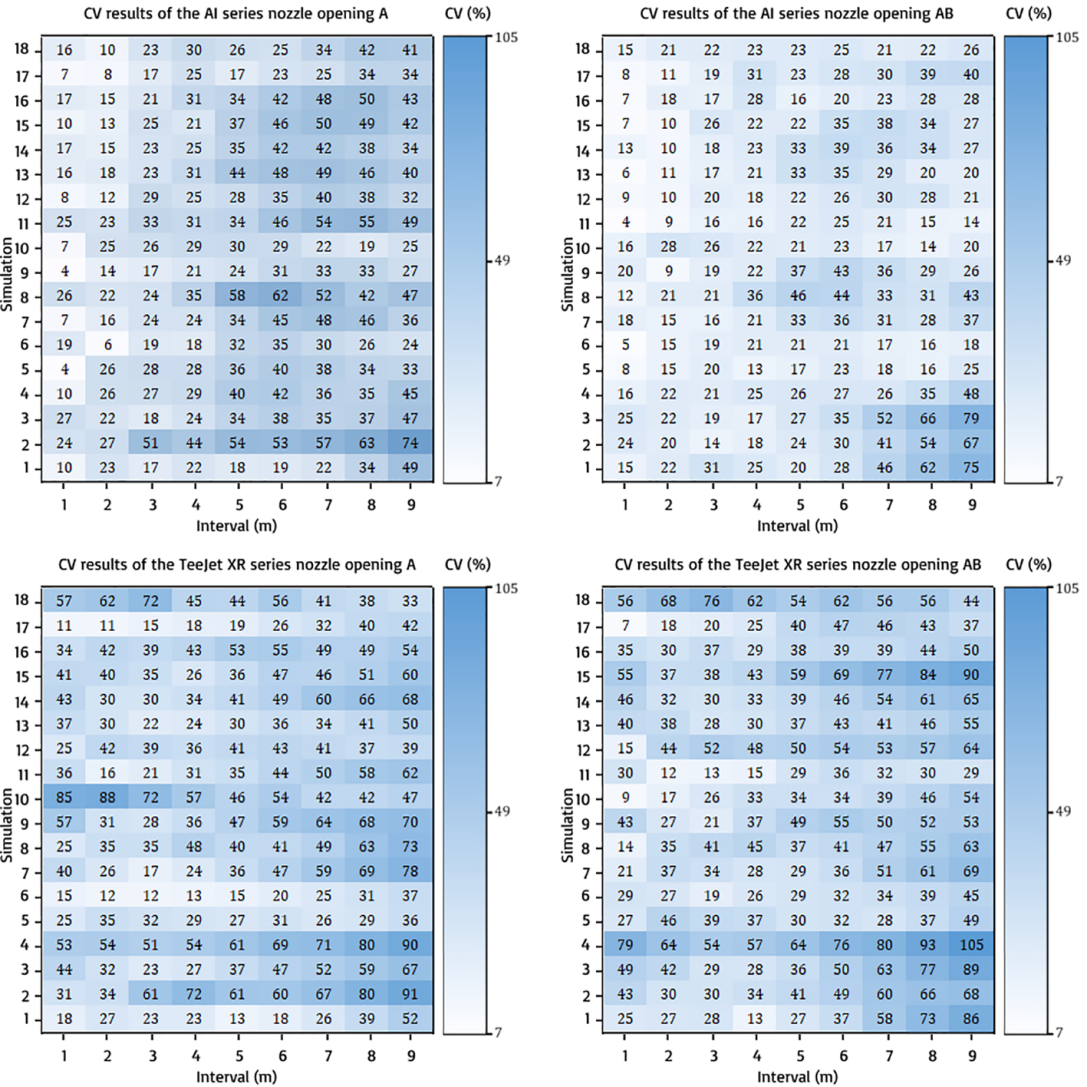
CV results of race track spraying pattern on all nozzle types and openings.

The race track spraying pattern exhibited good CV values at narrow intervals. **Figure 9** shows the CV results using the race track spraying pattern. AI series nozzle opening A in the resulting graph indicates that the CV value was good up to an interval of 4 m under some conditions. However, the CV of opening AB on the AI series nozzle type is<30% at high intervals of up to 9 m under some conditions. The CV results generated from the TeJeet XR series nozzle opening A indicate very rare CV values of<30%, which were even below standards under some conditions. However, the CV result of opening AB on TeeJet XR series, with CV values of<30% at scattered intervals, as in the simulation results of TJAB23123, where the standard CV was obtained at intervals of 1–5 m and 8–9 m.

#### 3.2.2 CV value of back-and-forth spraying pattern at 1–9 m intervals

**Figure 10:**
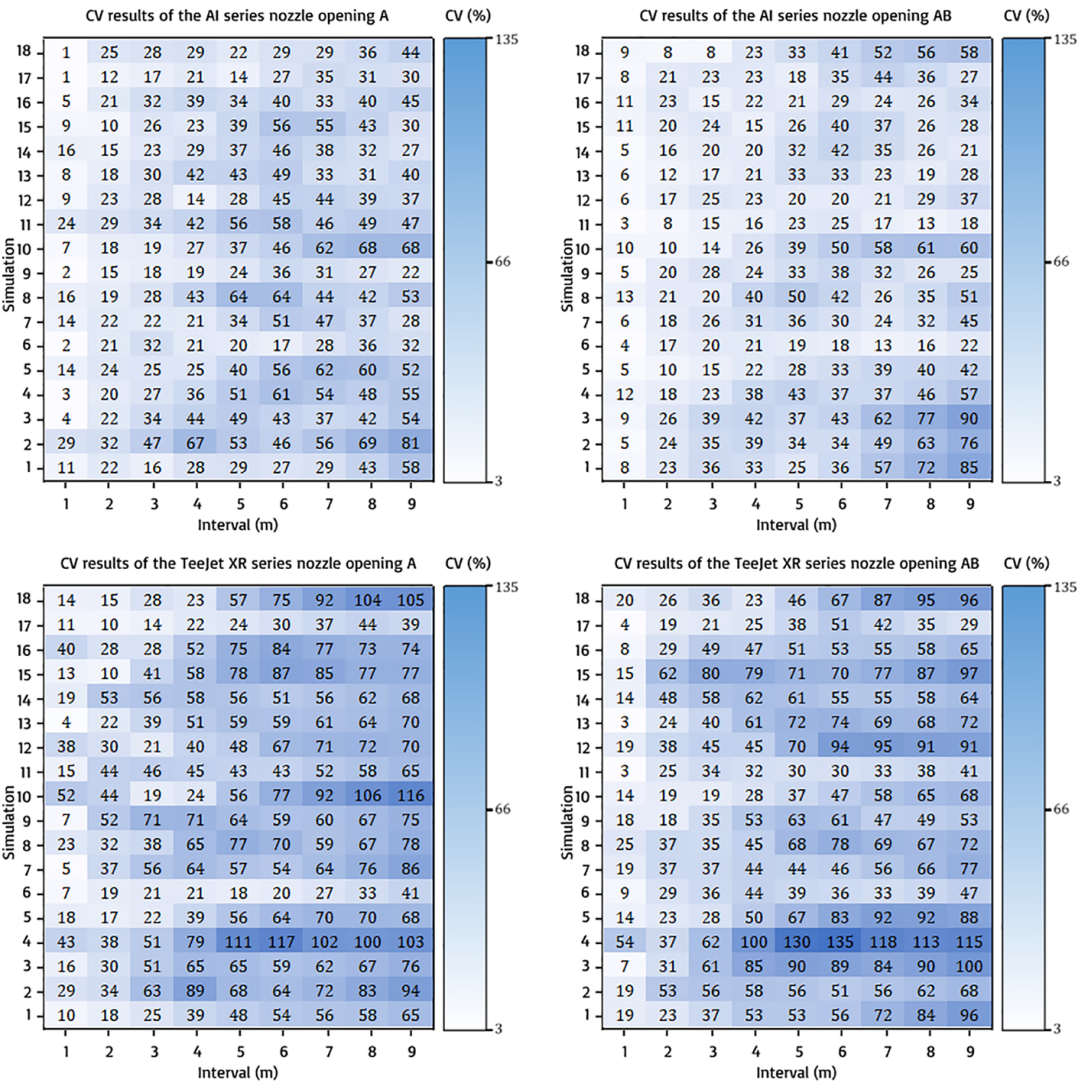
CV results of back-and-forth spraying pattern on all nozzle types and openings.

The back-and-forth spraying pattern exhibited improved distribution with CV values of<30% for pump opening A on both nozzle types than the race track spraying pattern, as shown in **Figure 10**. With pump opening A, the AI nozzle showed CV values of<30% up to an interval of 6 m under some conditions and exhibited CV values of<30% up to 9 m for both openings. The Tejeet XR series nozzle showed good CV values under wide intervals of up to 7 m with pump openings A under some conditions. An interesting phenomenon was observed in the AB pump opening with the Tejeet XR series nozzle exhibited a CV value of<30%, which is very rare and even tends to be narrower than that of A pump opening, from 1–3 m.

### 3.3 ANOVA analysis to validate the contribution of each parameter to the CV results

Based on the CV results on all range intervals, the variance analysis of each parameter was performed by merging all the data using the output CV value. The results revealed that parameters with a P-value of less than 0.05 exhibited a high relationship with the CV value. In contrast, parameters with a P-value above 0.05 were considered not to influence the CV value. **Supplementary Tables 1****–****4** show the results of the ANOVA tests conducted on each nozzle type and pump opening type. In **Supplementary Table 1**, which is the ANOVA of the AI series nozzle opening A, only the single parameter of wind direction has a P-value >0.05, and the parameter combination between RPM and flight was the only one that did not exert on the CV values (P-value >0.05). While in the AI series nozzle opening AB presented in **Supplementary Table 2**, only the parameter combination of wind direction and flight speed had a P-value >0.05. Referring to **Supplementary Table 3**, which analyzes the TeeJet XR series nozzle opening A, wind direction as a single parameter and its combination with flight speed has a P-value >0.05. In the ANOVA results for the TeeJet XR series nozzle opening AB represented in **Supplementary Table 4**, only the single parameter RPM and its combination with flight speed had a P-value >0.05. In this condition, wind direction significantly influenced the formation of CV values.

In addition, the combination of parameters in generating CV values was determined from the three-parameter levels used in the simulation. Some parameter combinations that have a P-value >0.05 produced similar CVs at each parameter level. Similar CV values, even with different levels, indicate that the combination of parameters does not have an extra impact on CV establishment. Most of these indications were generated by the wind direction and RPM parameters. The wind direction parameter has a zero value at one of its levels. In contrast, the RPM parameter had settings that were not constant in the simulation because it was done manually by the pilot. These two circumstances allow the combination of wind direction and RPM parameters to not highly influence the establishment of CV values.

### 3.4 Modeling of the control system based on the CV results

**Figure 11:**
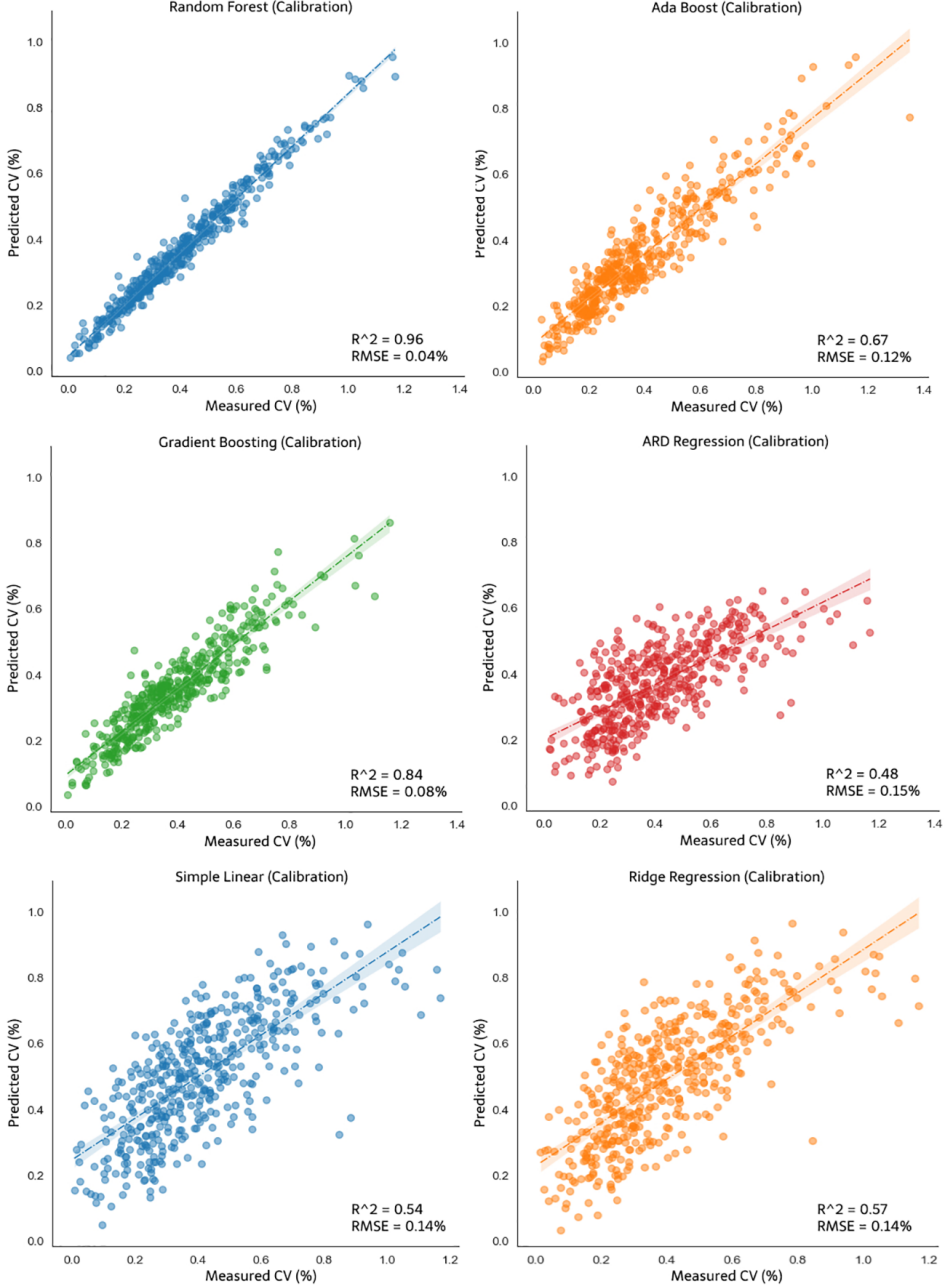
Calibration of the nonlinear models: random forest, ada boost, gradient boosting and ARDR, and linear models: simple linear and ridge regression for CV opening A.

**Figure 12:**
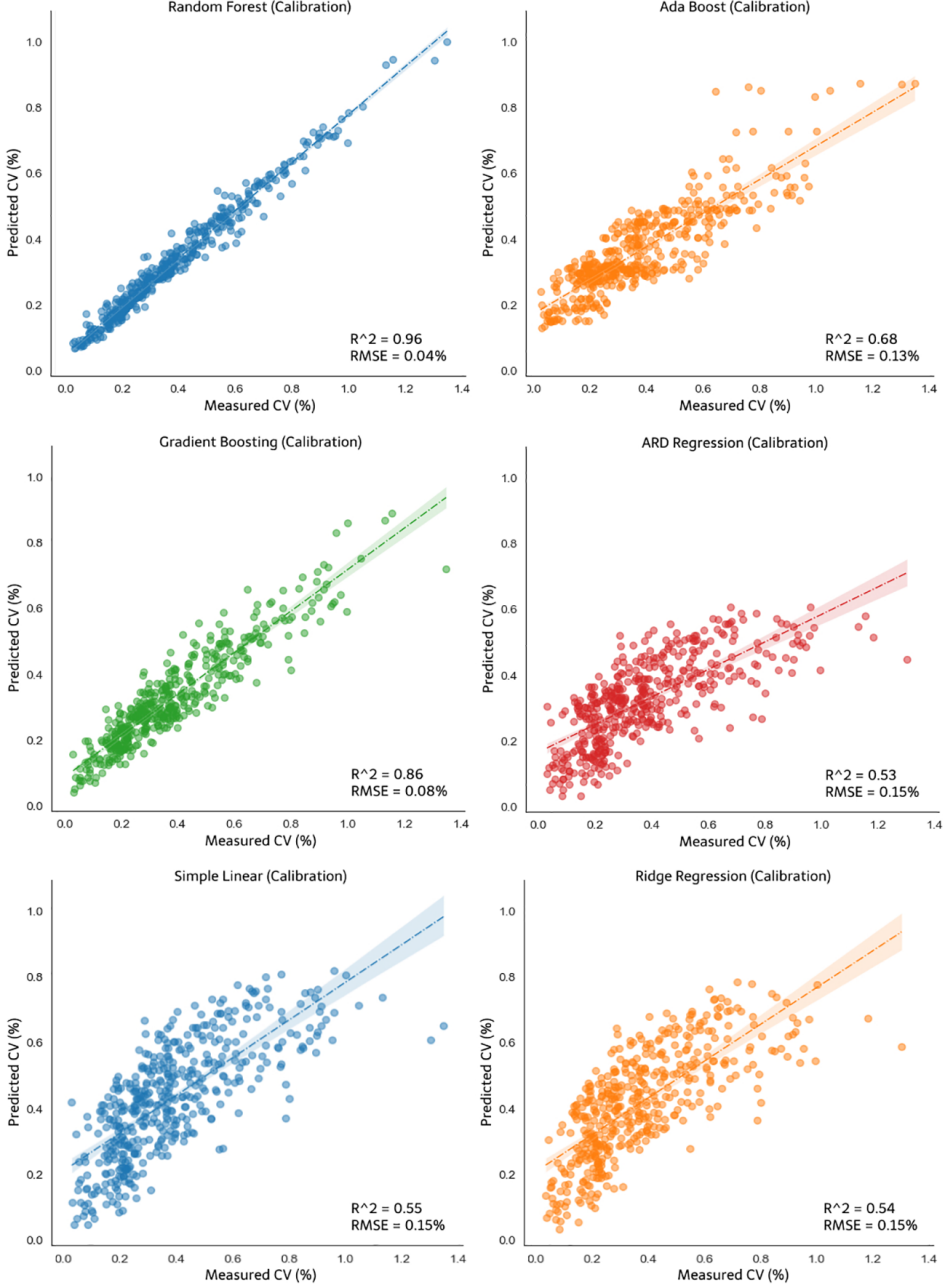
Calibration of the nonlinear models: random forest, ada boost, gradient boosting and ARDR, and linear models: simple linear and ridge regression for CV opening AB.

Obtaining data on the effects of variables on the output value is one of the crucial steps in developing the control system model; if the parameters that have been determined have a low level of influence on the output, then model development will be futile. Thus, the effects of the parameters of the CV value were confirmed, and the model was developed using each of the aforementioned methods. The following are the models generated from the six types of modeling used in this study. Modeling was divided into two for the CV output of each pump opening during simulation. The system control model was developed using four types of nonlinear models (random forest regression, ada boost, gradient boosting, and ARDR) and two types of linear models (simple linear regression and ridge regression). Modeling was performed for the different pump openings because both were actuator options. The y-axis and x-axis in **Figures 11**, **12** represent the predicted CV and actual measured CV, respectively. The predicted values were used to plot the predicted fit line to the actual measured CV values. Each figure below has an R-squared value representing the prediction accuracy, and the RMSE represents the prediction error. **Figure 11** shows the results of nonlinear regression and linear regression calibration for CV opening A, and **Figure 12** shows the result for CV opening AB. The random forest model exhibited the best accuracy (R^2 =^ 0.96) and the lowest error value (RMSE = 0.04%) for both A and AB pump openings. The R^2^ values of the ARDR model for pump openings A and AB were 0.48 and 0.53, respectively, the lowest among the three machine learning models, with RMSE of 0.15% for both pump openings, which was larger than those of the others. Under the linear regression models, the ridge regression model exhibited an R^2^ value of 0.57 for pump opening A, which was slightly higher than that of the simple linear regression for the same pump opening. However, both exhibited the same error values for pump openings A (RMSE = 0.14%) and AB (RMSE = 0.15%).

**Figure 13:**
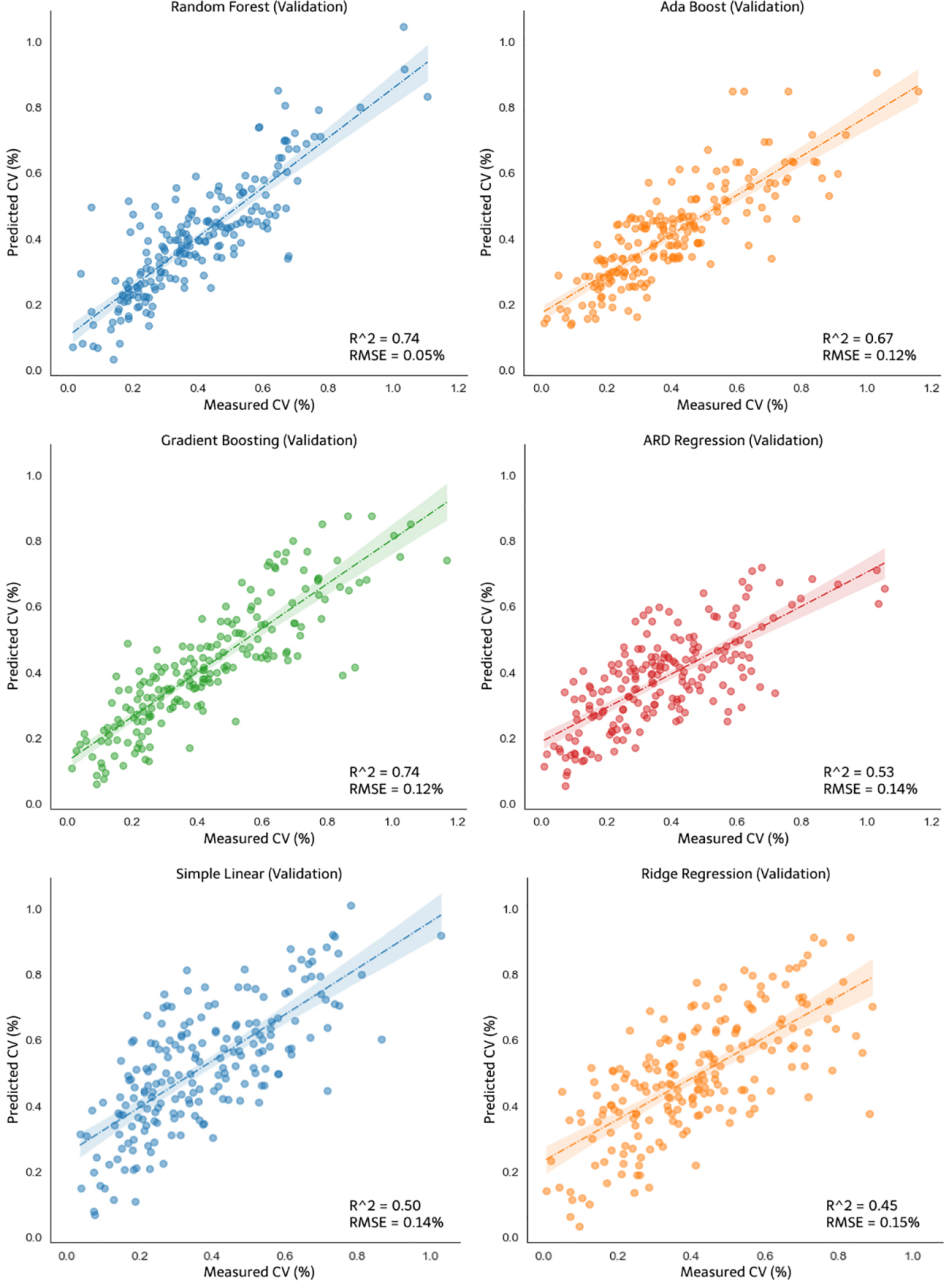
Validation of the nonlinear models: random forest, ada boost, gradient boosting, ARDR, and linear models: simple linear and ridge regression for CV opening A.

**Figure 14:**
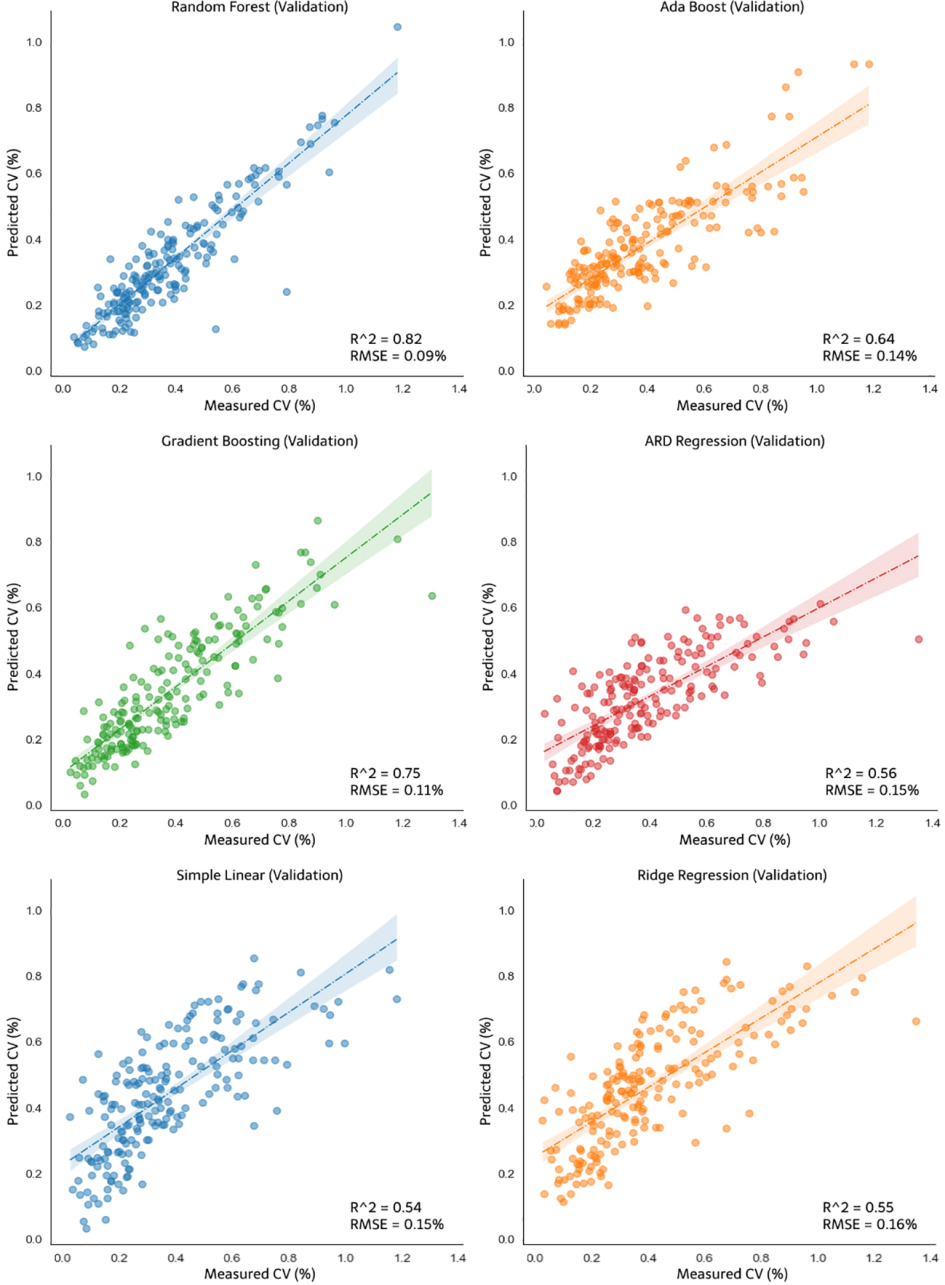
Validation of the nonlinear models: random forest, ada boost, gradient boosting, ARDR, and linear models: simple linear and ridge regression for CV opening AB.

The developed models were validated using 30% of the dataset not included in the model calibration dataset. **Figure 13** shows the validation result of nonlinear regression and linear regression for CV opening A, and **Figure 14** shows the validation result of the models for opening AB. The random forest model did not exhibit the best accuracy in the model validation result for CV opening A. The R^2^ value of the validation of the gradient boosting model for pump opening A was 0.74. Although this R^2^ value of the gradient boosting model was the same as that of the random forest. However, the RMSE of random forest remained the best among other validation models at 0.05%, which is smaller than that of gradient boosting (0.12%). In the validation for CV opening AB, as shown in **Figure 14**, the random forest still performed best compared to other models with an R^2^ value of 0.82 and RMSE of 0.09%. For the nonlinear models, the ARDR model exhibited the lowest R^2^ and RMSE values for pump opening A (0.53 and 0.14%, respectively) and pump opening AB (0.56 and 0.15%, respectively). The linear regression models did not exhibit improved R^2^ or RMSE values for calibration and validation compared to the random forest regression model. The simple linear and ridge regression exhibited poor R^2^ and RMSE values for model validation. The R^2^ and RMSE of the simple linear model for opening A were 0.5 and 0.14%, respectively, and those of the ridge regression model were 0.45 and 0.15%, respectively; for pump opening AB, the R^2^ and RMSE of the simple linear model were 0.54 and 0.15%, respectively, and those of the ridge regression model were 0.55 and 0.16%, respectively. Accordingly, the validation R^2^ value of each test process was generated, which strengthens the validity of the model that has been formed.

**Figure 15:**
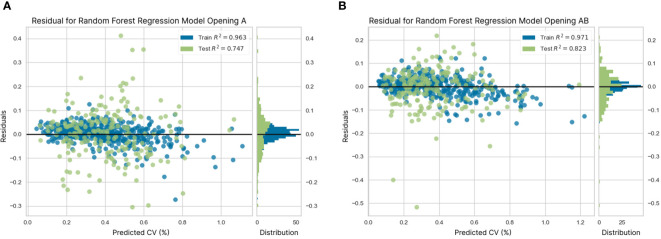
Distribution of residuals from random forest regression modeling **(A)** with pump opening A and **(B)** pump opening AB.

Among the models used to predict the CV values, the control system was modeled using the model with the best accuracy (R^2^) and error (RMSE) values (i.e., the random forest regression model). Particularly, the residual distribution values during calibration and validation are presented in **Figure 15**. The x-axis of the two figures illustrates the predicted CV value obtained during calibration and validation, and the error between the predicted value and the actual CV value is presented on the y-axis. **Figure 15A** shows the residual graph from the random forest regression modeling using pump opening dataset A. The error value was balanced, ranging from -0.3 to 0.4% CV. However, most errors occurred close to the CV number equal to 0, which illustrates an R^2^ of 0.96 for calibration and an R^2^ of 0.72 for validation. In **Figure 15B**, the prediction error value for the modeling of the pump opening AB exceeds -0.5% during the validation process. However, the R^2^ values for calibration and validation are higher than those in **Figure 15A**, where R^2 =^ 0.97 for calibration and R^2 =^ 0.77 for validation.

Python-based computational simulations were performed using a random combination of parameter values. This random combination was obtained using the orthogonal matrix method to obtain the most effective combination value from several levels of values of the eight parameters used, including flight speed, altitude, wind speed, wind direction, RPM, interval, spraying pattern, and nozzle. Each combination of parameter values that have been formed was used as a simulation condition for the random forest regression model for modeling both pump openings A and AB. Subsequently, both results of CV values from openings A and AB were used to provide a frame of reference in developing logic in the system to determine which pump opening can better fulfill the needs of the pesticides in the field. In this study, the CV value was first prioritized, where a value of less than 30% was considered as the best spray quality, whereas a value above 30% was assumed to be an unusable value. However, spraying must still be performed even if the two pumps cannot overcome the situation under certain conditions, so the second priority was applied in this circumstance to use the minimum amounts of pesticides possible to meet crop maintenance needs.

**Table 3** shows the simulation results conducted using the AI series nozzle under the spraying pattern race track, and the spraying distance was 8 m wide. The operating and environmental conditions were set using a speed of 3 m/s, flying height of 2 m, wind speed varied from 2.1 m/s, 2.7 m/s, 3.5 m/s, to 4.0 m/s, and random wind direction from 15° for minimum value to 45° for maximum value. **Table 3** indicates that the CV value from the pump opening model A and AB in condition number 15 was less than 30%, then the decision given is to only open pump A where this logic is the same as the first priority. In addition, in condition number 1, when the pump opening model A produced a CV of above 30%, and the AB opening model produced a CV of less than 30%, the decision given is to open the AB pump where the pump opening model A does not meet the priority criteria. The second priority regarding providing the minimum possible pesticide treatment can be seen in condition number 9, where both pump opening models produced CVs greater of above 30%, then the decision given is to open pump A. Practically opening pump A will provide a lower flow rate than opening both pumps and automatically, the pesticide is expelled at the minimum amount. **Table 4** shows the same simulation results when the XR series nozzle was used with a back-and-forth flight pattern at a spraying distance of 1 m under the same operating conditions and environment as the AI series nozzle simulation. Through the same logic commands as the XR series nozzle simulation, **Table 4** shows the following simulation results. For instance, in column number 1, when the CV of opening A exceeds 30% and that of opening AB is lesser, the decision given is to select pump opening AB. In all decisions that open only pump A, both actually had a CV value in the standard set, but when the state of both pump openings had a CV value of less than 30%, then the second priority applies, which is to choose the opening with a smaller flowrate value, namely pump opening A, to reduce the quantity of pesticide used. In accordance with both simulation results from **Tables 3** and **4**, the pump opening is in line with expectations in addressing the operating conditions of the UASS.

**Table 3:** Simulation results of random forest regression for AI series nozzle.

No.	Parameter condition								CV (%)		Decision
	FS (m/s)	Alt. (m)	WS (m/s)	WD (°)	RPM	Interval (m)	FM	Nozzle	A	AB	
1.	3	2	2.1	15	2900	8	2	1	32.8	25.7	A+B
2.	3	2	2.1	29	2900	8	2	1	32.8	25.2	A+B
3.	3	2	2.1	37	3100	8	2	1	43.6	29.3	A+B
4.	3	2	2.1	45	3100	8	2	1	43.6	29.3	A+B
5.	3	2	2.7	15	2900	8	2	1	32.8	25.7	A+B
6.	3	2	2.7	29	2900	8	2	1	32.8	25.2	A+B
7.	3	2	2.7	37	3100	8	2	1	43.6	29.3	A+B
8.	3	2	2.7	45	3100	8	2	1	43.6	29.3	A+B
9.	3	2	3.5	15	3100	8	2	1	39.9	30.1	A
10.	3	2	3.5	29	3100	8	2	1	40.1	29.0	A+B
11.	3	2	3.5	37	2900	8	2	1	26.6	23.8	A
12.	3	2	3.5	45	2900	8	2	1	26.6	23.8	A
13.	3	2	4.0	15	3100	8	2	1	39.9	30.1	A
14.	3	2	4.0	29	3100	8	2	1	40.1	29.0	A+B
15.	3	2	4.0	37	2900	8	2	1	26.6	23.8	A
16.	3	2	4.0	45	2900	8	2	1	26.6	23.8	A

**Table 4:** Simulation results of random forest regression for XR series nozzle.

No.	Parameter condition								CV (%)		Decision
	FS (m/s)	Alt. (m)	WS (m/s)	WD (°)	RPM	Interval (m)	FM	Nozzle	A	AB	
1.	3	2	2.1	15	2900	1	1	2	31.2	15.8	A+B
2.	3	2	2.1	29	2900	1	1	2	30.3	15.8	A+B
3.	3	2	2.1	37	3100	1	1	2	29.8	13.8	A
4.	3	2	2.1	45	3100	1	1	2	29.8	13.8	A
5.	3	2	2.7	15	2900	1	1	2	31.2	15.8	A+B
6.	3	2	2.7	29	2900	1	1	2	30.5	15.8	A+B
7.	3	2	2.7	37	3100	1	1	2	29.8	13.8	A
8.	3	2	2.7	45	3100	1	1	2	29.8	13.8	A
9.	3	2	3.5	15	3100	1	1	2	29.8	17.5	A
10.	3	2	3.5	29	3100	1	1	2	28.9	17.5	A
11.	3	2	3.5	37	2900	1	1	2	28.1	15.5	A
12.	3	2	3.5	45	2900	1	1	2	28.1	15.5	A
13.	3	2	4.0	15	3100	1	1	2	29.8	17.5	A
14.	3	2	4.0	29	3100	1	1	2	28.9	17.5	A
15.	3	2	4.0	37	2900	1	1	2	28.1	15.5	A
16.	3	2	4.0	45	2900	1	1	2	28.1	15.5	A

## 4 Discussion

Nozzle opening control systems to support the requirements of precision and uniform pesticide spraying in UASS were modeled based on the occurring parameters and are most influential in pesticide spraying operations. The determination of parameters was based on literature studies and UASS operating standard settings, both from nozzle type, pump pressure, pesticide dilution ratio, and spraying interval to UASS operating properties, such as flight speed, spray height, spraying pattern used, and fluid dynamics that occur, to produce sprays that must also meet standards, such as uniformity and precise quantity (Zhang et al., 2021). These conditions, particularly the UASS operating properties, cannot be determined based on user preferences. However, crop treatment operations must be adapted to the current needs in the field. In some cases, as reported by Martin et al. in 2019, the operation of UASS is highly influenced by weather conditions. They also analyzed the environmental conditions, particularly wind properties, that affect the results of the spray patterns and droplet spectra from UASS; thus, some are recommended to be kept from being operated in windy weather conditions (Martin et al., 2019), which are similar problems and phenomena that prompted this research.

The previously mentioned parameters were used as a reference in determining the parameters used in the simulation so that the environmental conditions can be adjusted according to the values of the predetermined parameters when using machine learning, where a dataset must be formed and used in the calibration and validation process. Obtaining CV values in this simulation requires a long data processing series and particular circumstances must also be discussed to validate this study. The rest of this chapter will go over the phenomena that occur during project development until there are variables that affect the output data. Furthermore, the discussion continues with the practical application of the results of this study to enable the evaluation of the practical application of the model to ensure its application in agriculture.

Various observations were made during the simulation, as shown in **Figure 8D**, where one of the simulations using the XR series nozzle produced an abnormal coverage value. A similar thing can happen in simulation when the operational conditions of the simulator are unstable. This specific kind of data can still be used because there is an R^2^ value in modeling that indicates the accuracy of the prediction when the dataset is used, as well as the accuracy of other machine learning methods (Wen et al., 2019) and this type of phenomenon contributes to the development of the model while considering a situation that may occur in actual use.

The ANOVA results revealed that all the parameters in calculating the CV value of each spraying pattern and interval range were valid with a P-value of less than 0.05 (Wang et al., 2023), indicating that the parameters significantly affected the output results (i.e., CV value). Few parameters, whether used as a single value or combined, have a P-value above 0.05. This begs the question of whether such parameters should still be used or whether they can be eliminated. RPM and wind direction are the only parameters with a P-value greater than the default. First, the parameters are discussed to discover the facts in the field. Almost all parameter values, except the RPM value, can be set precisely in the simulator. The use of optical sensors in calculating the number of rotor rotations does help in monitoring. Still, the rotor rotation was controlled manually, so the possibility of error is considerably high. Moreover, the wind direction parameter, which has a high P-value, indicates that this parameter does not really affect the output value because wind direction is directly affected by the wind speed value. If the wind speed value is zero, any predetermined wind direction value will not affect the output CV value. According to the orthogonal matrix used for the simulation, there are three levels of wind speed parameter, one of which is 0 m/s. This value will have no effect regardless of the wind direction value, which directly reduces the influence of the parameter on the CV.

Initially, the modeling in this study was performed separately for both nozzle types and spraying patterns used in the processing coverage data. However, these two classifications can be combined by assigning identities with numbers, increasing the data used in modeling. Recognizing that the number of datasets used influences the accuracy of the resulting model, this merging step was performed to improve the accuracy. Furthermore, as various investigations were performed, several modeling methods are still used as a comparative aspect where nonlinear or machine learning techniques have performed better than linear methods. The regression equation generated from four nonlinear methods also exhibited different performances, so random forest regression was selected as the modeling method with the best performance and was used for the development of the system control.

Using the simulator as a data collection platform had many advantages for this study. The predetermined parameters could be achieved with considerable ease and precisely. On the other hand, testing directly in the field would result in random parameter values that are difficult to establish. Developing an independent UASS spraying control system using machine learning is also an exciting advancement in precision agriculture. Achieving uniform spray distribution is one of the most critical factors in effective aerial spraying. The fact that the system processes data of CV values below 30% indicates that the machine learning algorithm can detect and correct uneven spray distribution. In addition, the amount of pesticide sprayed is another priority for developing precision spraying. Both scenarios improve the effectiveness of spraying and reduce the waste of chemicals, thus saving costs for farmers.

Another significant advantage of using machine learning for spraying control is the ability to target all UASS types in the future. This indicates that the system will be adaptable to a wide range of UASS, making it accessible to more farmers and agricultural businesses. Additionally, the ability to adapt and learn from new data means that the system can be continually improved and evolved over time, ultimately leading to even better results. However, there are also some potential challenges and considerations that should be addressed. One concern is the need for consistent and accurate data input, as the performance of the machine learning algorithm will depend heavily on the quality and accuracy of the data it receives. There also might be regulatory and ethical considerations regarding using autonomous UASS for spraying, particularly regarding safety and potential environmental impacts. Overall, developing an independent UASS spraying control system using machine learning is a promising advancement in agriculture technology, and it has the potential to improve spraying effectiveness, reduce waste, and increase cost savings for farmers. As the system is completed and ready to be fully examined, field tests will be conducted to determine the control system’s performance in addressing environmental conditions and the operation of the UASS. However, carefully considering potential challenges and ethical implications will be important moving forward.

## 5 Conclusions

The overall objective of this study was answered during the experiments. The essential results of this study are briefly summarized in the following conclusion:

Spraying distribution data was collected under the simulator environment conditions with 72 simulations. Using the spraying pattern and interval from 1–9 m, 648 kinds of CV datasets were obtained for each pump opening. AI nozzle produced a better ability to overcome drift at 1–7 m intervals under several simulation conditions and exhibited a CV value of<30%. The TeeJet nozzle type XR exhibited a narrower interval width with a CV of<30%, which ranges from 1–3 m in some simulation conditions. The CV values obtained in the spraying pattern race track tend to be greater, ranging from 4 to 26% at a spray interval of 1 m using the AI series nozzle and 7 to 57% at the XR series TeeJet nozzle. In contrast, in the back-and-forth spraying pattern, the CV values obtained ranged from 1 to 24% at a spray interval of 1 m while using the AI series nozzle and 3 to 54% at a spray interval of 1 m while using the XR series TeeJet nozzle. The control system was modeled using machine learning and linear regression methods based on the CV datasets for each pump opening. Using nine levels of interval parameters and two spraying pattern types also helps increase the number of datasets. In this study, the random forest regression model achieved the best accuracy and intercept error value compared to other models: it exhibited an R^2^ of 0.96 and RMSE of 0.04% for pump openings A and AB. This model could predict the CV value under parameters outside the modeling boundary. As confirmed in the simulation, the model can predict the CV value and make decisions on pump operation. The findings of this study confirmed the ability of random forest regression to develop a model for a functioning control system to establish an independent precision spraying control system.

## Data availability statement

The original contributions presented in the study are included in the article/**Supplementary Material**. Further inquiries can be directed to the corresponding author.

## Ethics statement

Written informed consent was obtained from the individual(s) for the publication of any potentially identifiable images or data included in this article.

## Author contributions

Conceptualization, AH, and XH. Methodology, AH, XH, S-HY, CH, and SB. Analysis, AH, S-HY, CH, and SB. Visualization, AH, S-HY, CH, and SB. Supervision, XH. Reviewing the manuscript, XH, S-HY, CH, SB, C-GL, D-HL, and YK. All authors contributed to the article and approved the submitted version.

## Supplementary material

Supplementary Figure 1 Indoor flight simulator for spraying distribution testing with **(A)** UASS mounted into the simulator and spraying on **(B)** water-sensitive paper layout.

Supplementary Figure 2 Positioning of the fan as a wind generator in the simulator.

Supplementary Figure 3 Control command used to control the device during the simulation.

Supplementary Figure 4 Weather station device with **(A)** ultrasonic anemometer and **(B)** data acquisition systems displaying wind property values.

Supplementary Figure 5 The image processing device for analyzing the spraying distribution data recorded on the WSP.

Supplementary Table 1 ANOVA of AI series nozzle opening A for single parameter and combination of parameters.

Supplementary Table 2 ANOVA of AI series nozzle opening AB for single parameter and combination of parameters.

Supplementary Table 3 ANOVA of TeeJet XR series nozzle opening A for single parameter and combination of parameters.

Supplementary Table 4 ANOVA of TeeJet XR series nozzle opening AB for single parameter and combination of parameters.
