# Supplementary figures and images for: Modeling of the control logic of a UASS based on coefficient of variation spraying distribution analysis in an indoor flight simulator
Source: Front Plant Sci. 2023 Aug 21;14:1235548. doi: 10.3389/fpls.2023.1235548 (PMC10475723; doi:10.3389/fpls.2023.1235548)

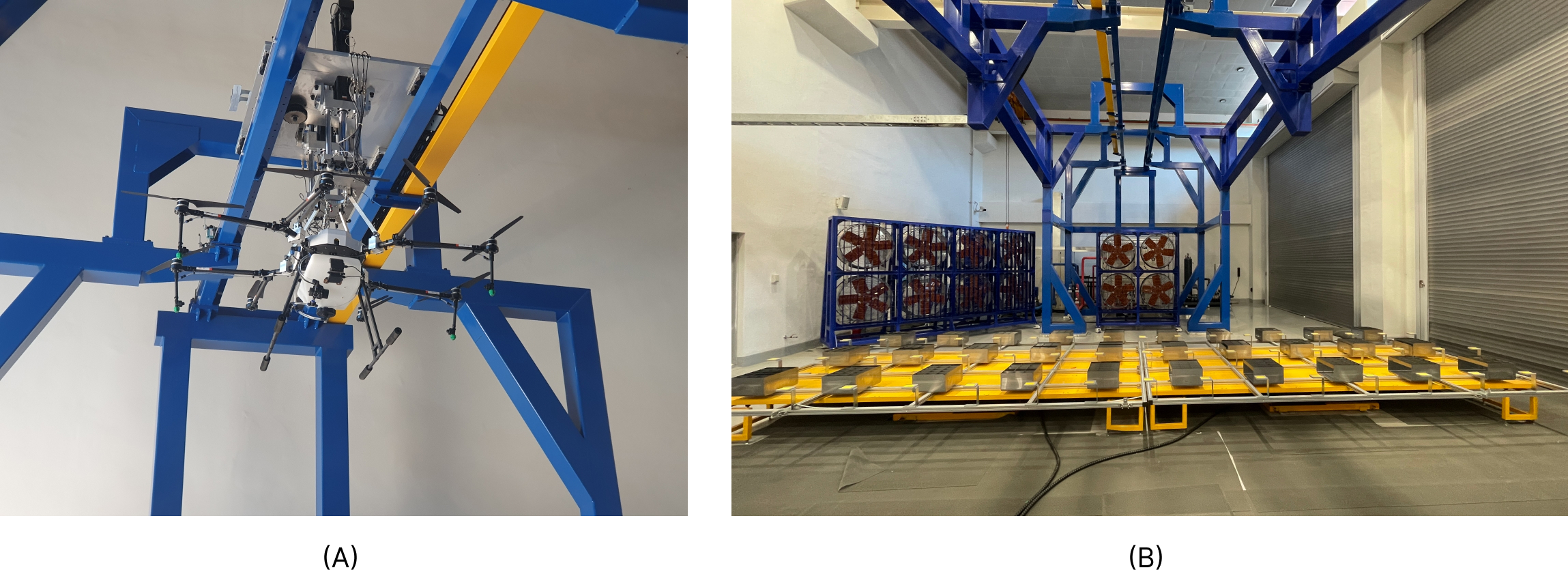

Supplement: Supplementary Figure 1 — Indoor flight simulator for spraying distribution testing with (A) UASS mounted into the simulator and spraying on (B) water-sensitive paper layout. [file Image_1.jpeg]

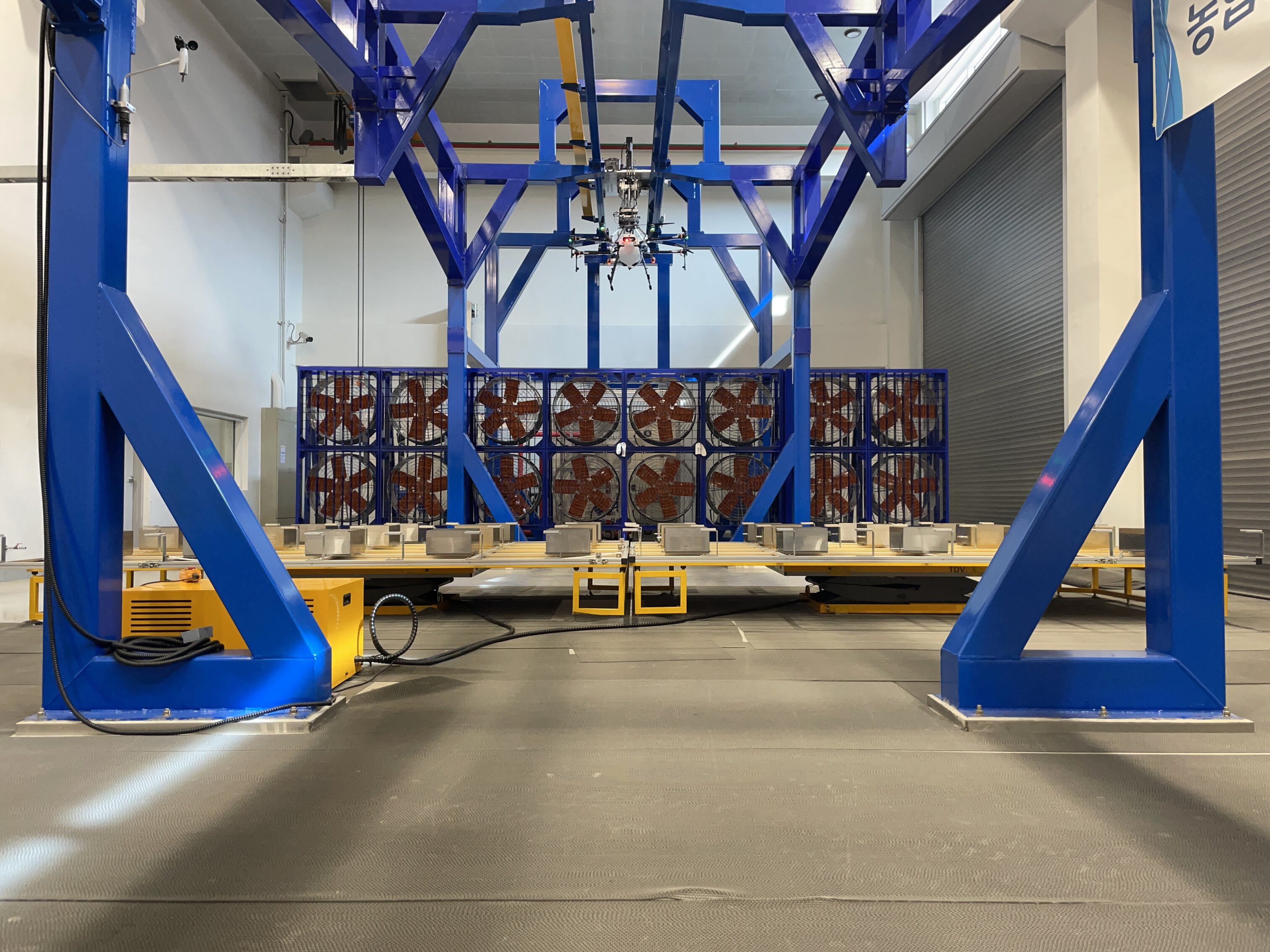

Supplement: Supplementary Figure 2 — Positioning of the fan as a wind generator in the simulator. [file Image_2.jpeg]

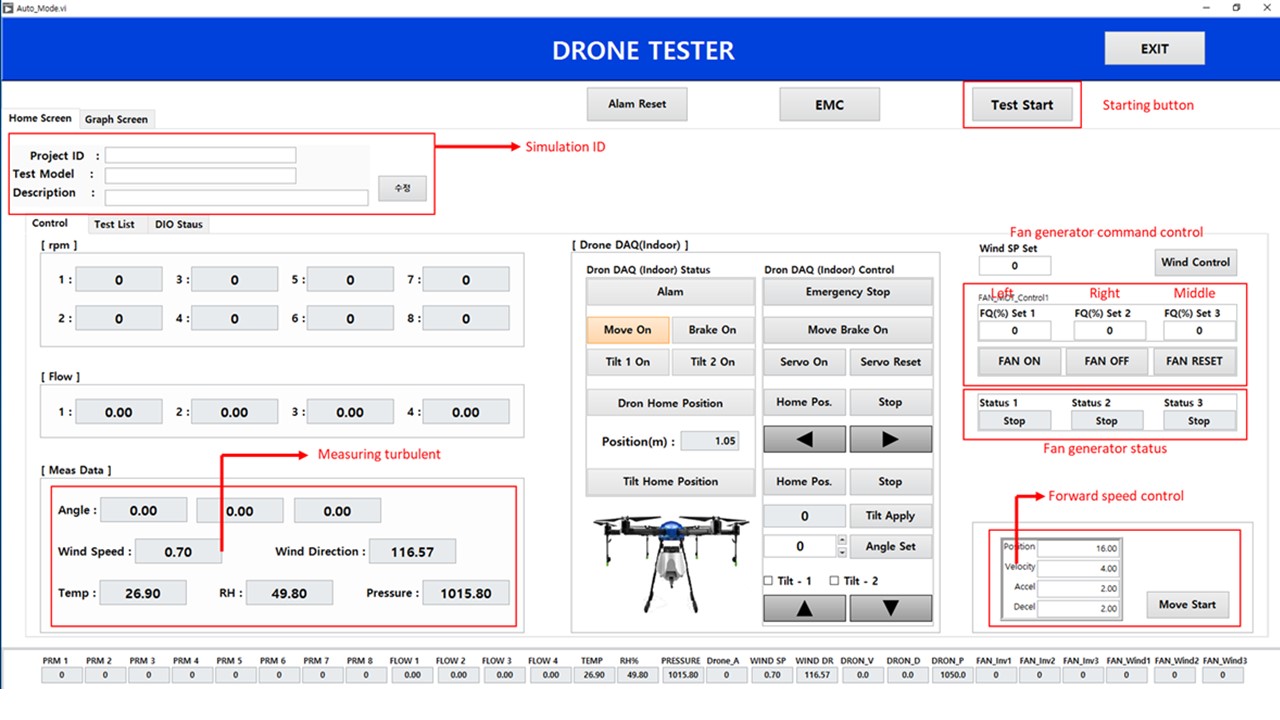

Supplement: Supplementary Figure 3 — Control command used to control the device during the simulation. [file Image_3.jpeg]

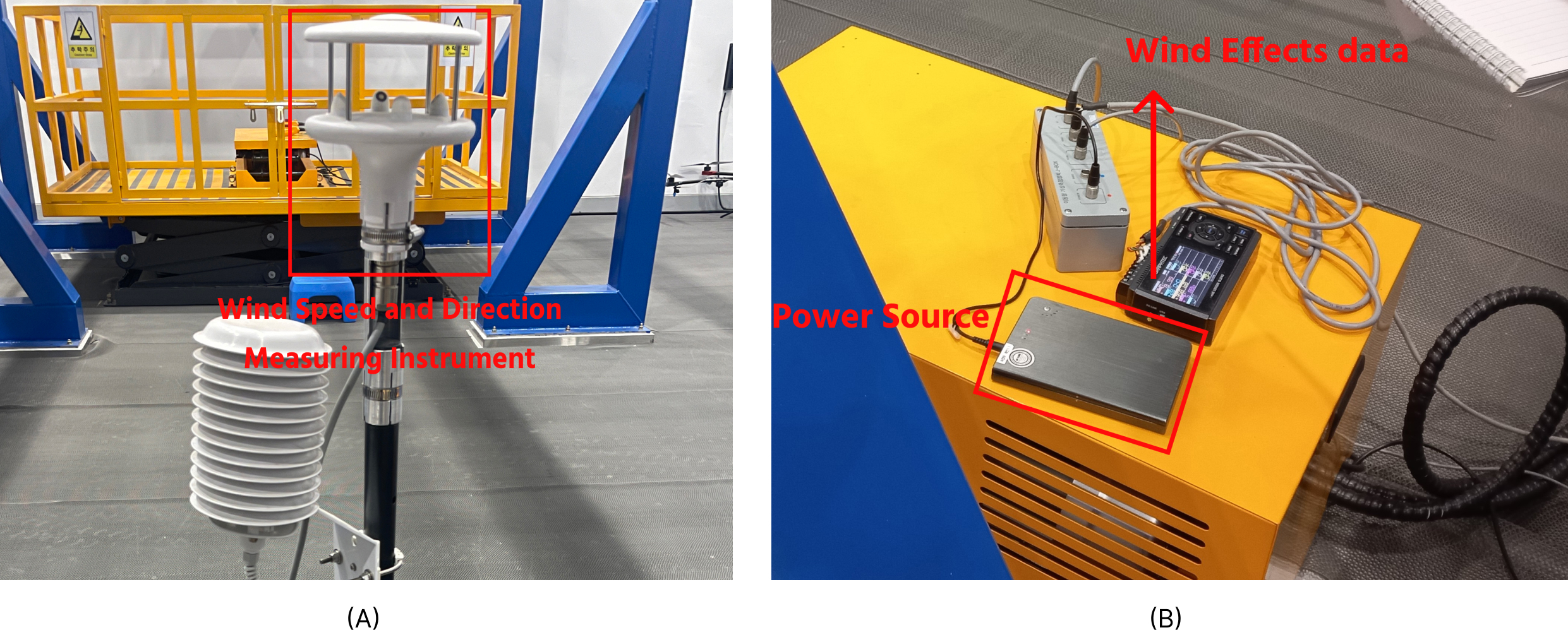

Supplement: Supplementary Figure 4 — Weather station device with (A) ultrasonic anemometer and (B) data acquisition systems displaying wind property values. [file Image_4.jpeg]

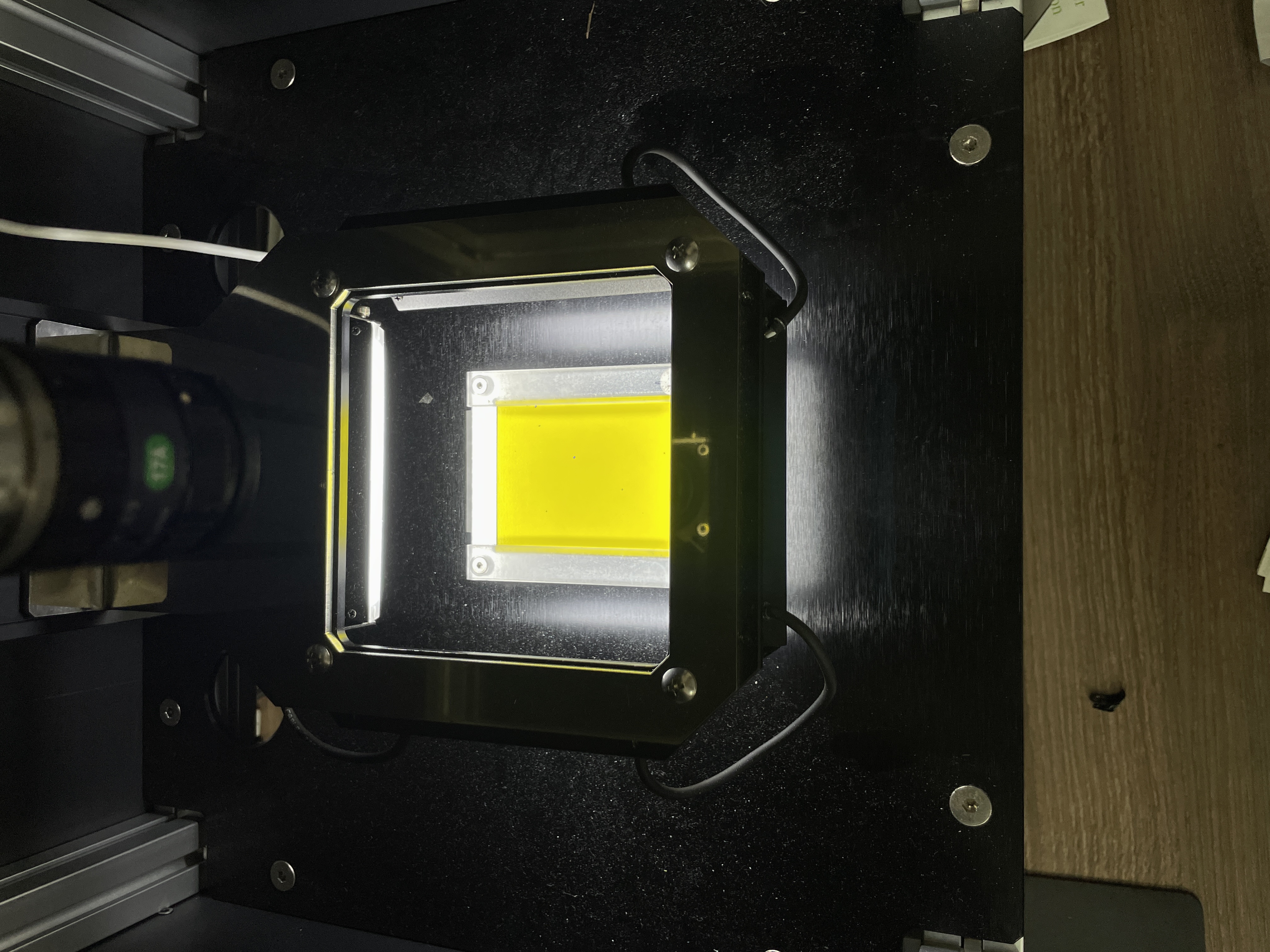

Supplement: Supplementary Figure 5 — The image processing device for analyzing the spraying distribution data recorded on the WSP. [file Image_5.jpeg]
