# Supplementary material for: Modeling of the control logic of a UASS based on coefficient of variation spraying distribution analysis in an indoor flight simulator
Source: Front Plant Sci. 2023 Aug 21;14:1235548. doi: 10.3389/fpls.2023.1235548 (PMC10475723; doi:10.3389/fpls.2023.1235548)
Supplement: Supplementary Table 1 — ANOVA of AI series nozzle opening A for single parameter and combination of parameters. [file DataSheet_1.docx]

Supplementary Table 1.

| Parameter | Correlation | | | | | |
| --- | --- | --- | --- | --- | --- | --- |
|  | Single | Flight Speed | Altitude | Wind Speed | Wind Direction | Interval |
| Flight Speed | 5.09×10^-04^ | - | - | - | - | - |
| Altitude | 1.24×10^-06^ | 7.24×10^-18^ | - | - | - | - |
| Wind Speed | 2.88×10^-02^ | 8.66×10^-26^ | 2.53×10^-19^ | - | - | - |
| Wind Direction | 1.59×10^-01^ | 1.07×10^-03^ | 2.23×10^-14^ | 1.62×10^-15^ | - | - |
| RPM | 4.89×10^-14^ | 1.17×10^-01^ | 8.85×10^-15^ | 2.94×10^-15^ | 3.81×10^-22^ | - |
| Interval | 2.51×10^-55^ | 1.62×10^-60^ | 9.93×10^-57^ | 7.89×10^-50^ | 3.47×10^-47^ | - |
| Flight Pattern | 9.09×10^-03^ | 1.73×10^-03^ | 1.02×10^-02^ | 3.95×10^-02^ | 2.43×10^-02^ | 9.84×10^-40^ |
|  | PR(>F) | PR(>F) | PR(>F) | PR(>F) | PR(>F) | PR(>F) |

Supplementary Table 2.

| Parameter | Correlation | | | | | |
| --- | --- | --- | --- | --- | --- | --- |
|  | Single | Flight Speed | Altitude | Wind Speed | Wind Direction | Interval |
| Flight Speed | 5.21×10^-07^ | - | - | - | - | - |
| Altitude | 7.78×10^-11^ | 4.39×10^-28^ | - | - | - | - |
| Wind Speed | 6.27×10^-03^ | 2.94×10^-05^ | 1.10×10^-12^ | - | - | - |
| Wind Direction | 2.28×10^-03^ | 8.84×10^-01^ | 1.53×10^-12^ | 7.98×10^-17^ | - | - |
| RPM | 9.07×10^-03^ | 1.37×10^-05^ | 1.98×10^-08^ | 2.54×10^-09^ | 2.83^-05^ | - |
| Interval | 2.57×10^-37^ | 4.55×10^-44^ | 9.36×10^-32^ | 1.48×10^-33^ | 3.16^-25^ | - |
| Flight Pattern | 9.22×10^-04^ | 1.63×10^-04^ | 1.56×10^-03^ | 3.86×10^-03^ | 2.60×10^-03^ | 7.24×10^-27^ |
|  | PR(>F) | PR(>F) | PR(>F) | PR(>F) | PR(>F) | PR(>F) |

Supplementary Table 3.

| Parameter | Correlation | | | | | |
| --- | --- | --- | --- | --- | --- | --- |
|  | Single | Flight Speed | Altitude | Wind Speed | Wind Direction | Interval |
| Flight Speed | 2.90×10^-02^ | - | - | - | - | - |
| Altitude | 2.38×10^-17^ | 1.35×10^-20^ | - | - | - | - |
| Wind Speed | 4.33×10^-06^ | 1.10×10^-19^ | 1.89×10^-24^ | - | - | - |
| Wind Direction | 5.80×10^-01^ | 1.29×10^-01^ | 2.11×10^-05^ | 3.10×10^-27^ | - | - |
| RPM | 1.93×10^-05^ | 3.41×10^-01^ | 3.35×10^-14^ | 1.08×10^-12^ | 4.52×10^-05^ | - |
| Interval | 3.53×10^-31^ | 1.18×10^-28^ | 4.55×10^-30^ | 6.53×10^-29^ | 3.98×10^-17^ | - |
| Flight Pattern | 1.21×10^-08^ | 8.30×10^-08^ | 1.28×10^-08^ | 1.13×10^-08^ | 1.39×10^-05^ | 1.83×10^-29^ |
|  | PR(>F) | PR(>F) | PR(>F) | PR(>F) | PR(>F) | PR(>F) |

Supplementary Table 4.

| Parameter | Correlation | | | | | |
| --- | --- | --- | --- | --- | --- | --- |
|  | Single | Flight Speed | Altitude | Wind Speed | Wind Direction | Interval |
| Flight Speed | 6.88×10^-06^ | - | - | - | - | - |
| Altitude | 6.40×10^-08^ | 4.76×10^-26^ | - | - | - | - |
| Wind Speed | 6.48×10^-02^ | 8.81×10^-15^ | 1.95×10^-25^ | - | - | - |
| Wind Direction | 9.07×10^-03^ | 4.38×10^-12^ | 2.49×10^-18^ | 4.32×10^-13^ | - | - |
| RPM | 5.24×10^-01^ | 1.82×10^-01^ | 5.15×10^-10^ | 2.12×10^-21^ | 2.02×10^-06^ | - |
| Interval | 2.83×10^-30^ | 1.88×10^-36^ | 2.05×10^-36^ | 6.26×10^-30^ | 7.32×10^-21^ | - |
| Flight Pattern | 9.86×10^-07^ | 1.36×10^-09^ | 9.26×10^-09^ | 4.33×10^-07^ | 3.16×10^-05^ | 5.04×10^-30^ |
|  | PR(>F) | PR(>F) | PR(>F) | PR(>F) | PR(>F) | PR(>F) |
